# Systematic review and meta-analysis of physical activity interventions to increase elementary children’s motor competence: a comprehensive school physical activity program perspective

**DOI:** 10.1186/s12889-024-18145-1

**Published:** 2024-03-15

**Authors:** Jongho Moon, Collin A. Webster, David F. Stodden, Ali Brian, Kelly Lynn Mulvey, Michael Beets, Cate A. Egan, Lori Irene Flick McIntosh, Christopher B. Merica, Laura Russ

**Affiliations:** 1 Department of Human Performance and Health Education, Western Michigan University, Kalamazoo, MI USA; 2 Department of Kinesiology, Texas A&M University - Corpus Christi, Corpus Christi, TX USA; 3 Department of Educational and Developmental Science, University of South Carolina, Columbia, SC USA; 4 Department of Psychology, North Carolina State University, Raleigh, NC USA; 5 Department of Exercise Science in Arnold School of Public Health, University of South Carolina, Columbia, SC USA; 6 College of Education, Health and Human Sciences Movement Sciences, University of Idaho, Moscow, ID USA; 7 College of Education, Department of Physical Education and Exercise Science, Lander University, Greenwood, SC USA; 8 College of Health and Human Sciences, University of North Carolina-Wilmington, Wilmington, NC USA; 9 Indepedent Researcher, Unaffiliated, Wilmington, USA

**Keywords:** Whole-of-school approach, Child development, Fundamental motor skills, Physical education

## Abstract

**Background:**

Regular participation in physical activity (PA) benefits children’s health and well-being and protects against the development of unhealthy body weight. A key factor in children’s PA participation is their motor competence (MC). The comprehensive school physical activity program (CSPAP) framework offers a way to classify existing PA interventions that have included children’s MC development and understand the potential avenues for supporting children’s MC. However, there have been no systematic reviews or meta-analyses of PA interventions and their effects on the MC of elementary school children (aged 5–12 years) from a CSPAP perspective.

**Methods:**

This study was conducted in accordance with the Preferred Reporting Items for Systematic Review and Meta-Analysis (PRISMA) statement. We searched seven electronic databases (PubMed/Medline, Embase, ERIC, SPORTDiscus, CINAHL, Web of Science, and PsycINFO) for articles on 29 November 2021. The CSPAP framework was used to categorize the different intervention approaches. This review was registered with PROSPERO (CRD42020179866).

**Results:**

Twenty-seven studies were included in the review, and twenty-six studies were included in the meta-analysis. A wide range of PA intervention approaches (e.g., single component or multicomponent) within the context of the CSPAP framework appear to be promising pathways in enhancing children’s MC. The results of the aggregate meta-analysis presented that effect sizes for the development of MC from pre-and post- intervention ranged from moderate to large (Hedges’ *g* = 0.41−0.79). The analysis revealed that the predicted moderators, including study length, delivery agent, and study design, did not result in statistically significant moderate variations in MC outcomes. There was, however, considerable heterogeneity in study design, instruments, and study context, and studies were implemented in over 11 countries across diverse settings.

**Conclusions:**

This study uniquely contributes to the literature through its primary focus on the effectiveness of PA interventions on elementary children’s MC. This review emphasizes the importance of customizing CSPAP to fit the specific characteristics of each school setting, including its environmental, demographic, and resource attributes. The effectiveness of CSPAP, particularly its physical education (PE) component, is significantly enhanced when these programs are adapted to address the unique needs of each school. This adaptation can be effectively achieved through targeted professional teacher training, ensuring that PE programs are not only contextually relevant but also optimized for maximum impact in diverse educational environments. Researchers and practitioners should pursue how to effectively translate the evidence into practice to better conceptualize CSPAPs designed for children’s MC development.

**Supplementary Information:**

The online version contains supplementary material available at 10.1186/s12889-024-18145-1.

## Background

It is well established that physical activity (PA) is crucial for the healthy growth and development of children [1, 2]; however, many children are not sufficiently active. Globally, over 85% of children and adolescents are not meeting the World Health Organization’s (WHO) recommended PA guidelines [3]. These guidelines suggest that children and adolescents should engage in at least 60 min of moderate-to-vigorous (MV) PA daily [3]. This level of activity is considered essential for maintaining physical health, supporting development, and fostering overall well-being in young individuals. Motor competence (MC) plays a major role in children’s PA participation [4–8]. MC can be defined as the capability to perform a wide range of motor acts or skills and involves both locomotor (e.g., running, jumping, and skipping) and object projection (e.g., throwing, catching, and kicking) skills [9]. The development of MC during childhood is crucial for a healthy life since it allow individuals to successfully participate in lifetime physical activities [4, 7, 8]. According to Stodden et al. [7], the attainment of adequate PA and MC levels should be viewed using a developmental perspective. In other words, children with greater MC were observed to spend more time in moderate-to-vigorous PA [10], whereas those with less developed MC appeared less physically active [4, 6]. Longitudinal evidence suggests that having higher levels of MC during childhood is associated with being more physically active later in life [11–13]. Conversely, low MC is hypothesized to result in decreased participation in PA in middle to late childhood, thus leading to a negative spiral of disengagement from an active lifestyle [7, 8].

Developing children’s and adolescents’ MC is a primary goal of physical education (PE) and is considered foundational to promoting lifetime participation in PA [14, 15]. Particularly during the elementary school years, establishing a robust foundation in MC is crucial as it facilitates the transition to more specialized movement forms in organized games and sports [16, 17]. This foundational stage involves the development of fundamental movement skills (FMS), which encompass a variety of basic movement patterns including locomotor skills, objective control skills, and stability skills [18]. These skills are essential building blocks for more complex and specialized motor skills acquired later in life [9]. Regular involvement in context-specific and developmentally appropriate PA experiences is critical [18–20]. The development of MC does not occur “naturally” and requires sufficient practice and experiences to successfully apply essential skills in the various PA activities that require their application [20, 21]. However, focused programming to support children’s MC development is decreasing for school-aged children, in tandem with a downward trend in the prevalence of PE [22]. It therefore becomes vital to explore and learn from innovations in school-based programming, which can not only counteract the declining provision of PE but also present expanded opportunities for children to develop their motor skills.

Recently, there has been increased interest in what the Institute of Medicine in the United States called a “whole-of-school” approach to PA promotion in children and adolescents, in which PA opportunities are provided before, during, and after school through the support of school staff, families, and community partners [23]. The International Society for Physical Activity and Health (ISPAH) named whole-of-school PA one of eight investments that work for increasing PA [24]. McMullen et al. [25] provide an insightful analysis of whole-of-school PA initiatives undertaken in Finland, Ireland, Poland, and the United States. Common to these initiatives is a focus on multiple PA opportunities, contexts, and promotion agents that coalesce around a strong PE program and build upon it with additional PA. While much of the attention given to whole-of-school PA centers on the extent to which such an approach can support children’s attainment of 60 min of PA each day (in line with current guidelines) [26–29], the contribution of expanded PA opportunities to the development of children’s MC also warrants investigation. If designed appropriately, PA opportunities beyond PE may allow children to apply and practice what they learn in PE and continue to develop their motor skills [30].

A plethora of review studies have substantiated the beneficial impact of PA interventions on the enhancement of MC among children and adolescents, as evidenced by research such as Lorås [31] and Zeng et al. [32]. Notably, Barnett et al. [33] undertook a systematic review of longitudinal data pertaining to MC and health, elucidating the interplay between MC and health outcomes (e.g., weight status, health-related fitness). Complementing this, Han et al. [34] and Hassan et al. [35] independently deduced that exercise and PA interventions markedly improved FMS and motor coordination in children and adolescents, with aerobic activities showing pronounced efficacy in augmenting object control and gross motor skills. Ruggeri et al. [36] further corroborate this viewpoint, demonstrating that interventions focusing on motor skills and PA fostered enhanced participation, activity, and improvements in body structure and function in children diagnosed with autism spectrum disorder. Conversely, Jones et al. [37] present a caveat, highlighting that despite the established positive correlation between PA and motor skills in early childhood, the exact causative directionality of this relationship remains an area of ambiguity.

Overall, there is substantial evidence supporting an association between PA and MC, but less is known about the development of children’s MC in the context of whole-of-school PA approaches. The current review aims to bridge this existing knowledge gap by synthesizing and evaluating the collective impact of PA interventions while considering how these interventions align with whole-of-school PA promotion. For the purposes of this review, we have adopted the comprehensive school physical activity program (CSPAP) model as a representative whole-of-school PA framework. In the United States, the Centers for Disease Control and Prevention (CDC) named the CSPAP model as the national framework for school-based PE and PA [1]. The model includes five components: (a) quality PE, (b) PA during school (DS), (c) PA before and after school (BAS), (d) staff involvement (SI), and (e) family and community engagement (FCE) [1, 38]. This study will dissect the nuances of how various PA interventions, categorized under CSPAP components, distinctly influence MC outcomes in elementary school children. The purpose of this study, therefore, is to conduct a systematic review and meta-analysis, based on CSPAP framework, of the effectiveness of PA interventions in increasing the MC of elementary school children (5–12 years). By doing so, it endeavors to offer a refined perspective on PA’s role in enhancing MC, thereby setting the stage for more effective, tailored CSPAP program implementations in the future. Ultimately, this systematic review and meta-analysis seeks not only to consolidate the existing research but to push the boundaries further in understanding and optimizing the role of PA in the development of children’s MC.

## Methods

### Registration and protocol

This study followed the Preferred Reporting Items for Systematic Reviews and Meta-Analyses (PRISMA) guidelines [39] with additional recommendations for systematic meta-reviews [40] and was registered with the International Prospective Register of Systematic Reviews at https://www.crd.york.ac.uk/prospero/ (registration number CRD42020179866).

### Inclusion/eligibility criteria

Studies with the following characteristics were included in our review:

1. Participants were aged 5–12 years (primary/elementary school);
2. PA interventions primarily focused on improving and assessing MC/FMS components;
3. Type of interventions: Any school-, home-, or community-based interventions for children with clear intent to improve MC/FMS proficiency;
4. Type of studies: Employed a Cluster-Randomized Controlled Trials (C-RCTs) design, RCTs, or rigorous (matched or statistically controlled) quasi-experimental design.

### Exclusion criteria

Studies with the following characteristics were excluded from the review:

1. Studies that reported on a population of focus outside of the age range defined above; or participants who were not ‘typically developing’ (i.e., had a clinically diagnosed physical or intellectual disability or condition affecting movement, e.g., autism, visual impairment, cerebral palsy, traumatic brain injury/concussion);
2. Studies that did not aim to improve and assess at least one of MC/FMS components outcomes were excluded;
3. Studies reported as abstracts, theses/dissertations and unpublished literature were excluded.

It should be mentioned that our search was not limited by the CSPAP framework, since the framework incorporates all conceivable circumstances and opportunities for PA promotion for children. Additionally, we aimed to include all relevant PA interventions regardless of whether the researchers used the CSPAP framework explicitly in their published publications. Thus, we did not perform our search using the phrase “CSPAP” or variants of the terms (e.g., comprehensive PA, whole-of-school PA).

### Search strategy and terms

The studies were obtained on November 29, 2021 using seven electronic databases: PubMed/Medline, Embase, ERIC, SPORTDiscus, CINAHL, Web of Science, and PsycINFO. The search strategy consisted of four elements: study population (e.g., elementary school student), study design, intervention (e.g., PA and exercise), and outcome measures (e.g., MC; see detailed search strategy in Supplementary Table 1). The search was limited to peer-reviewed academic journal articles published in English in all available years.

### Data extraction/collection process

Data were imported into Endnote X9.3 (The Thomson Corporation Corp, Stanford,

CT, USA) and duplicates were removed. The selected references were imported to a web-based software platform that streamlines the production of systematic reviews (Covidence systematic review software, Veritas Health Innovation, Melbourne, Australia, available at www.covidence.org). This first level of screening, two independent reviewers (CAE and CBM) screened the titles and abstracts of retrieved records for possible inclusion. Of the records identified as possibly eligible, the full texts were obtained, and two independent reviewers (LF and LR) assessed the records for inclusion. For each included study, two reviewers (CAE and LF) extracted data into a pre-defined Microsoft Excel (Microsoft Corporation, Redmond, WA, USA) data collection form. Data were extracted on the following: type of study design; the intervention approach, based on the CSPAP framework; the sample size; the intervention characteristics including session duration, frequency, length, delivery, and the name of programs; the types and methods of measured outcomes including the specific instrument; the fidelity of implementation measure; and the main results. For all steps in the screening process and data extraction, a third reviewer (JM or CAW) checked the data for errors, and discrepancies were resolved through discussion and consensus of judgement. If data were missing, authors were contacted.

### Qualitative data synthesis

Extracted results showed information including the article reference, study design, intervention approach (i.e., CSPAP components used), study characteristics (country, school setting, school level, name of the intervention program, participants, intervention deliverer, and MC outcomes), dose, main results, and implementation fidelity reporting. Results were organized into three sections by: (1) study design (i.e., C-RCT/RCT and N-RCT), (2) interventions addressing a single CSPAP component (i.e., PE, PADS, PABAS, and FCE) and (3) interventions addressing multiple CSPAP components (e.g., PE + 1 additional component and PABAS + FCE).

### Quantitative data synthesis

Effect sizes were calculated for the intervention group relative to the comparison group for each study. When the necessary data were not available in the original article, we requested it from the authors. If data could still not be obtained, we extracted the data from the graphs when available. If that was not possible, we excluded the study from the quantitative analysis. A meta-analysis for a given MC outcome was conducted if at least three studies reported interventions addressing the same CSPAP components and provided sufficient data for the calculation of effect size.

Pre- and post-intervention mean ± standard deviation (SD) for a given MC outcome, and sample size from each study were converted to Hedges’ *g* effect size [41]. Specifically, we calculated standardized mean differences both for outcome scores at the end of the intervention period (post-intervention) and change-from-baseline (pre-intervention) outcomes. Scores post-intervention effect sizes refer to intervention group results compared with comparison or control group results after interventions. We did not include follow-up assessment data. In all analyses, we used the random-effects model to account for differences between studies that might impact the treatment effect [42, 43]. The effect size values are presented alongside their respective 95% Confidence Intervals (CIs). Calculated effect sizes were interpreted using the following scale: small (*g* < 0.40), moderate (*g* = 0.40−0.70), and large (*g* > 0.70), according to the Cochrane Handbook [44]. Heterogeneity (i.e., between studies variability) was evaluated using the I-squared (*I*^2^) statistic. *I*^2^ values of < 25%, 25−75%, and > 75% were considered to represent low, moderate, and high levels of heterogeneity, respectively [45]. The risk of bias was explored using the visual inspection of funnel plots and Egger’s regression test [46]. Publication bias was not produced as the meta-analyses included < 10 studies/interventions [47].

A series of models were analyzed to address the following: (1) the pooled effect of PA interventions across all studies on elementary school age-children’s MC (overall and by measurement), (2) the pooled effect of interventions using only PE compared to the pooled effects of other single-component interventions that did not use PE (PADS only, PABAS only, and PADS + PABAS + FCE) on children’s MC, and (3) the pooled effects of interventions using PE plus additional CSPAP components (PE + 1 and PE + 2) on children’s MC. In addition, moderation analyses were performed to explore the impact of potential explanatory variables and moderators (intervention duration [< 6 months vs. ≥ 6 months], delivery agent [research team vs. school-based team vs. combined], and study design [C-RCT/RCT vs. N-RCT]) on the effect sizes with meta-regressions when sufficient data were available (i.e., at least ten studies for each explanatory variable) [44]. The results were expressed as regression coefficients estimates, 95% CIs and the p-value. All analyses were carried out using the Comprehensive Meta-Analysis program (version 3.3.070; Biostat, Englewood, NJ, USA). The statistical significance threshold was set at *p* <.05.

### Risk of bias assessment

Risk of bias in the included studies was assessed by two reviewers (JM and CAW) independently through discussion using the Cochrane Risk of Bias Tool (RoB 2.0) with additional considerations for C-RCTs and RCTs [48], which consists of five domains and an overall judgment [40]. The five domains are: (1) bias arising from the randomization process; (2) bias due to deviations from the intended interventions; (3) bias due to missing outcome data; (4) bias in measurement of the outcome; and (5) bias in selection of the reported result. Based on the answers (yes, probably yes, probably no, no, not applicable, no information) to a series of signaling questions in the guidance document, the judgment options within each domain consist of “low risk of bias,” “some concerns”, or “high risk of bias” [48].

The N-RCT (i.e., quasi-experimental) studies were assessed with the Risk of Bias in Non-randomized Studies of Interventions (ROBINS-I) tool [49], which consists of seven domains and an overall judgement. The seven domains are: (1) bias due to confounding; (2) bias in selection of participants into the study; (3) bias in classification of interventions; (4) bias due to deviations from intended intervention; (5) bias due to missing data; (6) bias in measurement of outcomes and (7) bias in selection of the reported result [49]. Domain-specific risk of bias assessment was used to judge the overall risk of bias for each study. Disagreements between reviewers were resolved through discussion and consensus by a third evaluator (CAE). Before correcting for observed differences, the agreement between reviewers was assessed using a Kappa correlation for risk of bias (κ > 0.8). A risk of bias graph was made via the *robvis* R package [50].

## Results

**Fig. 1:**
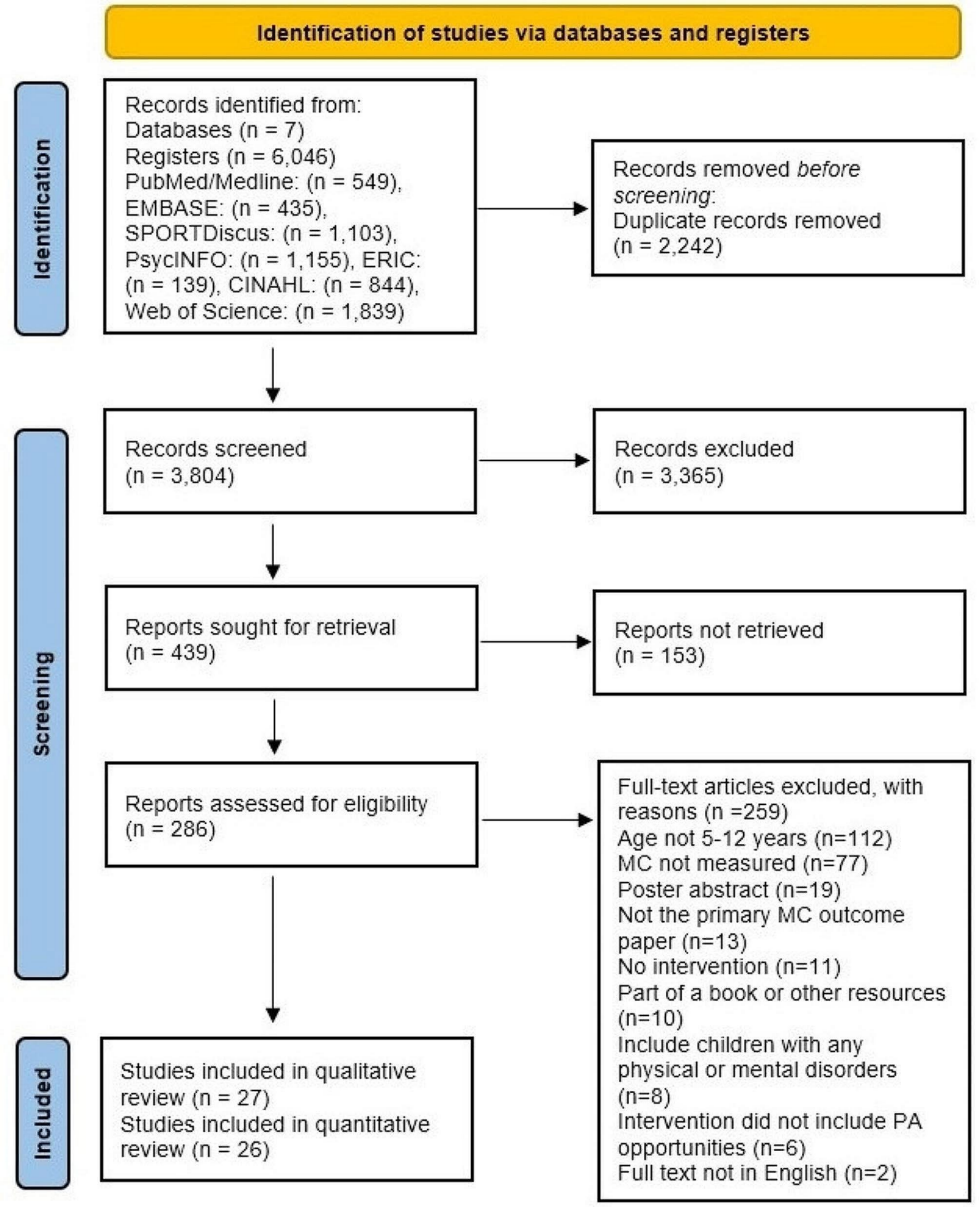
PRISMA flow chart of the search process of screened, included, and excluded articles

A total of 6,064 search records were initially identified. The authors screened 3,804 records after removing duplicate records. This first level of screening, separated by title and abstract, identified 439 full-text articles to be reviewed for eligibility. Ultimately, of the remaining 286 articles, 27 studies were included in the qualitative synthesis and 26 studies were included in quantitative synthesis. The process of literature identification and selection is outlined in the PRISMA flowchart (Fig. 1). The quality assessment for C-RCTs or RCTs revealed five studies as low risk in quality, four studies as having some concerns in quality, and one study as high risk (Supplementary Fig. S1). For N-RCTs, nine studies were evaluated as low risk of bias, six studies as moderate quality, and two studies as serious risk of bias (Supplementary Fig. S2). Generally, the studies included a lack of clear description of randomization procedures and lack of clarity regarding drop-out rates. There are some studies that did not assess the fidelity of the interventions to determine if they were implemented as intended. Additionally, most of the studies had some concerns due to deviations from the intended interventions.

### Characteristics of the included studies

Across the 27 studies, there were a total of 13,281 participants (49% female, 51% male) from 306 classes and 191 schools. The sample size ranged from 13 [51] to 4,234 participants [52] with the age of intervention children ranging from five to 12 years. Ten studies were conducted in North America (i.e., Canada and United States), seven in Europe (i.e., Finland, Germany, Ireland, Netherlands, Poland, and United Kingdom), eight in Australia, one in Asia (i.e., China), and one in South America (i.e., Brazil). Additionally, four studies were conducted in urban settings, one in rural and urban settings, one in a rural setting, and two in a suburban setting. The setting was not specified in 19 of the studies. The detailed characteristic of all of the included studies in Supplementary Appendix Fig. S1.

### Descriptions of CSPAP components

#### Study design

Eight C-RCTs (30%), two RCTs (7%), and 17 N-RCT studies (63%) were included in this review.

### C-RCT/RCT

Ten studies were C-RCTs or RCTs [53–63], with the average number of schools and classes across all studies being 14 (range 1–91) and 33 (range 2–157), respectively [57, 58]. The average sample size was 369 students (range 28–1,736), [57, 63] and the total number of students was 3,054 (48% Female, 52% Male). The average intervention duration, frequency, and length was 30 weeks (range 5–96) [58, 59], two times per week, and 55 min per session (range 15–120) [54, 63], respectively. For measurement of children’s MC, five studies of ten (50%) used Test for Gross Motor Development (TGMD-2nd Edition or -3rd Edition) [53, 54, 58, 61, 62], one study (10%) used Körper-koordinationtest Für Kinder (KTK) [55], two studies used other measurements (e.g., Dordel-Koch-Test [DKT]; 20%) [57, 63], and two studies (11%) did not specify a measurement tool [59, 60].

#### N-RCT

17 studies were N-RCTs (i.e., quasi-experimental), with the average number of schools and classes across all studies being four (range 1–9) [52, 64–66] and six (range 2–20) [65, 67, 68], respectively. The average sample size was 564 students (range 13–1,460) [51, 69], and the total number of students was 3,686 (49% Female, 51% Male). The average intervention duration, frequency, and length was 30 weeks (range 4–176) [70, 71], two times per week (range 1–5) [51, 52, 67, 72], and 59 min per session (range 25–120) [51, 67, 70, 73], respectively. For measurement of children’s MC, 11 studies (64%) used TGMD-2 or -3 [64, 65, 67–69, 72–77], four studies (24%) used KTK [51, 65, 66, 71], two studies used other measurements (e.g., PE Metrics; 11%) [62, 78], and one study (5%) did not specify a measurement tool [70]. Two studies used TGMD-2 and KTK [65, 67].

### Single CSPAP component interventions

Considering the CSPAP framework, nine studies out of 18 (50%) used only PE for the intervention [52, 53, 58, 62, 65, 67, 71, 73, 78], three studies (17%) used only PADS [57, 70, 75], four studies (22%) used only PABAS [60, 64, 72, 76], and two studies (11%) used only FCE [55, 63]. No study used only SI for the intervention.

#### PE

For the studies that used only PE as the intervention approach, the average number of schools and classes across all studies were four (range 1–9) [52, 58, 65] and seven (range 2–20) [58, 65, 67], respectively. The average sample size was 691 students (range 42–4,234) [52, 58], and the total number of students was 3,054 (50% Female, 50% Male). The average intervention duration, frequency, and length was 20 weeks (range 4–96) [52, 71], two times per week, and 57 min per session (range 25–120) [67, 73], respectively.

PE interventions involved PE lessons that incorporated revised FMS activities [73]; movement activities related to specific motor skills [53]; a PE curriculum that included motor skill themes and physical fitness activities [52]; goal-directed learning [71, 78]; a movement program (Brain Gym) involving a series of simple-to-challenging FMS intended to enhance cognitive processing, psychomotor and whole-brain learning [58]; the Professional Learning for Understanding Games Education (PLUNGE) program, which aimed to increase the complexity of challenges experienced through gameplay-situated learning for the improvement of FMS [62]; and a gymnastics curriculum developed by Gymnastics Australia, which aimed to develop stability, locomotor and object control skills, and general body coordination [65, 67]. The intervention deliverer varied across interventions. One study (11%) was delivered by a research team [58], seven studies (78%) by a school-based team (i.e., Trained PE teachers, classroom teachers and students) [52, 53, 65, 67, 71, 73, 78] and one study (11%) by a combined team (e.g., research team, school-based team, and parents) [62]. Additionally, four studies (44%) reported fidelity of intervention using observation and/or checklists [52, 53, 62, 67].

#### PADS

For the studies that used PADS as the single intervention component [57, 70, 75], the average number of schools and classes across all studies being 35 (range 7–91) and 84 (range 11–157, respectively [57, 75]. The average sample size was 826 students (range 336–1,736) [57, 75], and the total number of students was 2,479 (55% Female, 45% Male). The average intervention duration, frequency, and length was 81 weeks (range 20–176) [57, 62, 70, 75], two times per week, and 33 min per session (range 15–60) [57, 75], respectively.

PADS interventions involved structured games to increase children’s FMS [75]; short daily classroom exercises [57]; and a whole-of-school health promotion approach aimed to develop children’s FMS by modifying the physical and social environment [70]. Two studies (75%) were delivered by a school-based team [70, 75] and one study (25%) by a combined team [57]. None of the studies reported fidelity of intervention.

#### PABAS

For studies that used only PABAS as an intervention approach, the average number of schools and classes across all studies was seven (range 1–16) [60, 64] and three [76], respectively. The average sample size was 63 students (range 31–146) [60, 64], and the total number of students was 252 (60% Female, 40% Male). The average intervention duration, frequency, and length was 13 weeks (range 8–26) [60, 64], three times per week (range 2–5) [60, 72], and 60 min per session, respectively.

The PABAS interventions involved outdoor low-organized games and indoor sports-based activities including swimming, floor hockey, and soccer as after school activities [72]; an after school program aiming to teach children the 12 basic motor skills from the TGMD-2 criteria [64, 76]; and an after school club program that included multi-games activities, which focused on FMS development by using offering numerous opportunities for practice with learning cues [60]. Three studies (75%) were delivered by a research team [60, 64, 76] and one study (25%) by an after school-based team (i.e., after school program leaders) [72]. Two studies (50%) reported fidelity of intervention (using field observations) [64, 76].

#### FCE

For studies that used FCE as the single intervention component, the average number of schools and classes across studies was one and two, respectively [55, 63]. The average sample size was 193 students, and the total number of students was 385 (49% Female, 51% Male). The average intervention duration, frequency, and length was 27 weeks (range 6–48), two times per week, and 60 min per session, respectively [55, 63].

The FCE interventions involved family involvement by providing tailored counseling [55], structured PA homework/materials, educating parents to an increase children’s MC, and goal-setting [55, 63]. One study (50%) was delivered by a research team (i.e., coaches and research assistants) [55] and one study (50%) by a combined team (i.e., research team and parents) [63]. Both studies (100%) reported fidelity of intervention using observation and checklists.

### Multiple CSPAP components interventions

A total of nine studies (33%) used intervention approaches that could be mapped onto multiple components within the CSPAP framework [51, 54, 59, 61, 66, 68, 69, 74, 77]. The most commonly used components in multicomponent approaches were PE (*n* = 8) followed by SI (*n* = 7) and PADS (*n* = 5). PABAS (*n* = 3) and FCE (*n* = 3) were included in less than half of the multicomponent studies. One multicomponent intervention did not include a PE component. No study included all five CSPAP components.

#### PE + 1 additional CSPAP component

Three of the studies (33%) reported an intervention that included PE + 1 additional CSPAP component. Two studies included SI and one study included PABAS. The average number of schools and classes across all studies were three (range 1–7) [59, 61] and 21 (range 2–56) [59, 68], respectively. The average sample size was 202 students (range 31–467) [59, 68], and the total number of students was 605 (29% Female, 71% Male). The average intervention duration, frequency, and length was 50 weeks (range 6–96) [59, 61], three times per week (range 1–4) [61, 68], and 45 min per session (range 30–60) [59, 61], respectively.

PE + 1 interventions involved the Professional Learning for Understanding Games Education (PLUNGE) program that aimed to improve children’s FMS in PE lessons through a professional learning process involving classroom teacher education and mentoring [61]; a Sports, Play, and Active Recreation for Kids (SPARK) based PE program designed to enhance children’s motor skills through a classroom teacher professional development program [61]; and PE lessons combined with an extracurricular after school program (From Fun To Sport) with an emphasis on the development of children’s FMS [68]. All three interventions were delivered by a school-based team (i.e., PE teachers and trained classroom teachers). Only one study (33%) reported fidelity of intervention using lesson observations [61].

### PE + 2 additional CSPAP components

Three studies (33%) reported interventions that included PE + 2 additional CSPAP components. Two studies included the combination of SI and PADS with PE [69, 77], and one study included SI and FCE with PE [66]. The average number of schools and classes across all studies was two and three, respectively. The average sample size was 664 students (range 174–1460) [69, 77] and the total number of students was 1991 (50% Female, 50% Male). The average intervention duration, frequency, and length was 11 weeks (range 10–12), two times per week, and 50 min per session (range 30–60), respectively [66, 69, 77].

The PE + 2 interventions involved a CSPAP-based gross motor skill development program including the Dynamic PE for Elementary School Children curriculum during PE lessons, PA engagement opportunities throughout the school day during recess and regular classroom time (during which teachers integrated PA into academic lessons and classroom activity breaks via stretching, walking, jumping, or relaxation activities), and SI that provided teacher professional training to increase the quality of PE [69]; the Great Leaders Active StudentS (GLASS) program that included trained students who instructed their peers to improve FMS during PE lessons and classroom settings, and trained teachers supporting their peers’ instruction, which contributed an SI component to the program [77]; and physical exercise sessions during PE lessons (e.g., circuit training, aerobic/sports activities, and recreational games), parent support to promote PA during after school classes, and nutritional education sessions (e.g., goal setting and dietary counselling with parents) [66]. One study (25%) was delivered by a school-based team (i.e., PE teachers, classroom teachers, and students) [77] and two studies (75%) by a combined team (i.e., PE teachers, classroom teachers, medical or healthcare staff, parents, PA leaders, and the research team) [66, 69]. Only one study (33%) reported fidelity of intervention using observations and a checklist [77].

### PE + 3 additional CSPAP components

Two studies (22%) reported interventions that included PE + 3 additional CSPAP components. One study included the combination of SI, PADS, and FCE with PE and one study included SI, PADS, and PABAS with PE. The average number of schools and classes across all studies was seven and 25, respectively [54, 74]. The average sample size was 667 students (range 357–976) and the total number of students was 1333 (44% Female, 56% Male) [54, 74]. The average intervention duration, frequency, and length was 42 weeks (range 36–48), three times per week (range 1–5), and 98 min per session (range 75–120), respectively [54, 74].

One PE + 3 intervention involved a CSPAP-based program that aimed to optimize the quality of PE. The intervention provided PA opportunities before and after school as well as during recess/lunch time, which created a number of opportunities for children to engage in free play or semi-structured PA by applying skills learned during PE lessons. Additionally, PA was integrated into academic lessons and classroom activities, and SI was addressed with continuous teacher training and assistance throughout the intervention [74]. The other PE + 3 intervention involved the implementation of six PA policies to support the promotion of PA and FMS competency within the PE lessons in combination with SI and PADS through teacher professional learning, student leadership workshops, and PA promotion tasks to achieve awards during recess and lunch. In addition, the intervention incorporated FCE via school–community connections (e.g., inviting local sporting organizations to assist with school sport programs) as well as a range of approaches targeting the home environment (e.g., newsletters, parent evening, and FMS homework) [54]. Both studies were delivered by a combined team (i.e., PE teachers, classroom teachers, principals, parents, PA leaders, and community leaders) and both reported the fidelity of the intervention using observations and a checklist [54, 74].

### Multicomponent interventions without PE

One study (11%) included the combination of PABAS and FCE with no PE component [51]. The total number of students was 13 (62% Female, 38% Male). The intervention duration, frequency, and length were ten weeks, one time per week, and 120 min per session, respectively [51]. The intervention program involved a community-based program with an additional home-based PA motor development program using goal-setting and parental motivation strategies [51]. The intervention was delivered by a combined team (i.e., researchers and parents) [51]. The study did not report fidelity of intervention.

### Meta-analysis

#### Effectiveness across all interventions

The meta-analysis for total 26 studies indicated a statistically significant and large pooled intervention effect on children’s total MC (Hedges’ *g* = 0.71; 95% CI = 0.60–0.81; *p* <.001; *I*^2^ = 78.4%; Supplementary Fig. S3). The relative weight of each study in the analysis ranged from 1.40 to 6.22%. For all included studies, Egger’s regression test for asymmetry of the funnel plot was not significant (β = 0.32, *p* =.17), indicating no evidence of publication bias (Supplementary Fig. S4). Results from the meta-regression found that intervention duration (β = -0.04; 95% CI = -0.29–0.19; *p* =.69), delivery agent (β = -0.22; 95% CI = -0.65–0.21; *p* =.31), and study design (β = 0.13; 95% CI = -0.10–0.36; *p* =.28) were not found to be a statistically significant moderator variables/factors affecting overall study effect sizes (i.e., children’s total MC).

In a subsequent analysis, the studies adopting the TGMD − 2 or -3 tests were compared to studies including other types of assessments. The latter analysis was conducted as a proxy for effects of types of MC measurements. 17 studies measured children’s MC using the TGMD-2 or -3 tool. The meta-analysis for studies indicated a statistically significant and large pooled intervention effect on children’s total MC (Hedges’ *g* = 0.79; 95% CI = 0.63–0.95; *p* <.001; *I*^2^ = 38.2%; Supplementary Fig. S5). The relative weight of each study in the analysis ranged from 3.88 to 9.30%. Additionally, 11 studies measured children’s MC using other measurement tools (e.g., KTK, PE Metrics, and DKT). The meta-analysis for these studies indicated a statistically significant and moderate pooled intervention effect on children’s total MC (Hedges’ *g* = 0.57; 95% CI = 0.42–0.72; *p* <.001; *I*^2^ = 64.1%; Supplementary Fig. S6). The relative weight of each study in the analysis ranged from 3.77 to 12.62%. Specifically, five studies measured children’s MC using KTK tool. The meta-analysis for studies indicated a statistically significant and moderate pooled intervention effect on children’s total MC (Hedges’ *g* = 0.41; 95% CI = 0.28–0.57; *p* <.001; *I*^2^ = 86.7%; Supplementary Fig. S7). The relative weight of each study in the analysis ranged from 4.04 to 12.82%.

### Effectiveness of PE only vs. other single component interventions

Nine studies used only PE as the intervention approach. The meta-analysis for these studies indicated a statistically significant and large pooled intervention effect on children’s total MC (Hedges’ *g* = 0.79; 95% CI = 0.55–1.04; *p* <.001; *I*^2^ = 66.7%; Supplementary Fig. S8). The relative weight of each study in the analysis ranged from 7.40 to 13.30%. Eight other studies used non-PE single component intervention approaches (i.e., PADS + PABAS + FCE). The meta-analysis for these studies indicated a statistically significant and moderate pooled intervention effect on children’s total MC (Hedges’ *g* = 0.48; 95% CI = 0.29–0.68; *p* <.001; *I*^2^ = 85.0%; Supplementary Fig. S9). The relative weight of each study in the analysis ranged from 10.81 to 24.62%.

In a subsequent analysis, the studies that used non-PE single component interventions were analyzed. Specifically, three studies used only PADS as the intervention approach. The meta-analysis for these studies indicated a statistically significant and moderate pooled intervention effect on children’s total MC (Hedges’ *g* = 0.48; 95% CI = 0.27–0.69; *p* <.001; *I*^2^ = 76.5%; Supplementary Fig. S10). The relative weight of each study in the analysis ranged from 14.20 to 45.38%. Additionally, three studies used only PABAS as the intervention approach. The meta-analysis for these studies indicated a statistically significant and moderate pooled intervention effect on children’s total MC (Hedges’ *g* = 0.50; 95% CI = 0.13–0.89; *p* <.05; *I*^2^ = 0.0%; Supplementary Fig. S11). The relative weight of each study in the analysis ranged from 25.28 to 46.18%.

### Effectiveness of interventions addressing multiple CSPAP components

Three studies reported intervention approaches that used PE and one additional CSPAP component (PE + 1) to increase children’s MC. The meta-analysis for these studies indicated a statistically significant and moderate pooled intervention effect on children’s total MC (Hedges’ g = 0.64; 95% CI = 0.33–0.95; *p* <.001; *I*^2^ = 48.2%; Supplementary Fig. S12). The relative weight of each study in the analysis ranged from 13.25 to 18.30%. Additionally, three studies reported intervention approaches that used PE and two additional CSPAP components (PE + 2) to increase children’s MC. The meta-analysis for these studies indicated a statistically significant and moderate pooled intervention effect on children’s total MC (Hedges’ *g* = 0.55; 95% CI = 0.27–0.82; *p* <.001; *I*^2^ = 52.9%; Supplementary Fig. S13). The relative weight of each study in the analysis ranged from 12.98 to 32.75%.

## Discussion

The purpose of this systematic review and meta-analysis was to use the CSPAP framework to synthesize the evidence of the effectiveness of PA interventions in increasing MC as a primary outcome of children aged 5–12. Twenty-seven studies met the inclusion criteria and were included in the qualitative analysis and twenty-six studies were included in the quantitative analysis. The results of the aggregate meta-analysis indicate that effect sizes for the development of MC from pre-post intervention ranged from moderate to large. In light of our results, a wide range of CSPAP-aligned PA intervention approaches appear to be promising avenues in enhancing children’s MC. However, there is considerable variation in study design, sample size, delivery agent, and study context, and studies were implemented in over 11 countries across diverse settings. Additionally, the results do not show clear evidence that increased PA duration or frequency (i.e., dose) has a detrimental effect on the development of children’s MC, which aligns with McDonough et al. [79].

Results of this review indicated that the majority of studies included PE as a component of either a single (33%) or multicomponent (30%) approach and showed beneficial effects on the development of children’s MC. Specifically, PE commonly included the integration of movement activities with cognitively challenging PA learning experiences related to FMS and implementation of an established curriculum (e.g., SPARK) with professional teacher training [52, 59, 61]. Especially in PA interventions that employ complex, challenging learning tasks, how such activities are delivered and implemented may crucially affect learning outcomes [80]. Jiménez-Díaz et al. [81] in a review of 36 articles, present that naturally occurring PE classes were less effective at increasing children’s MC than a research specialist-led motor intervention, based on PE teachers’ lack of expertise for designing and implementing developmentally appropriate movement activities. However, our results showed that school-based teams (i.e. PE teachers and classroom teachers) can play a crucial role in increasing children’s MC with professional training and structured curriculum. Likewise, ongoing teacher training and support appears to be a key element of effective PE curriculums and successful interventions by enhancing the unique features of qualitative enrichment [82–84].

The PE-based programs often evaluated outcomes related to PA, fitness, and body composition [85]. Conversely, most of the included studies focused on the development of MC beyond PA opportunities. These results are consistent with those of a previous systematic review, which found that FMS-based intervention programs appeared to have larger effects than interventions focused strictly on increasing PA [86]. Further, when it comes to curriculum, research has demonstrated and noted the importance of structure when promoting children’s motor skill development [20, 87]. In this review, a number of the PE components within the CSPAP framework assessed an enhanced PE curriculum with a focus on optimal MC development as compared to traditional PE or free play [53, 62], some simply tested the benefit of an additional time allotment of PE [68], and some compared both modified PE and time spent in PE lessons [71, 78]. Overall, implementing a purposefully designed intervention approach with PE lessons had a positive effect on the development of MC. Further, of those studies that compared PE intervention programs versus typical PE [70], results support the importance of the quality of instructional approaches that enable students to have developmentally appropriate tasks/activities with learning cues, multiple opportunities for individual practices in a mastery climate, and individualized feedback [88–91]. These results were quite similar to those demonstrated by Morgan et al. [92], who highlighted the benefits of using a pedagogical approach to develop children’s FMS in PE.

In this review, we considered dose (i.e., as the amount of time/duration devoted to motor skill instruction and practice) [93], specifically < 6 months vs. ≥ 6months, as a possible moderating factor in the effectiveness of PA interventions on MC development in children. Based on the results, however, the intervention dosage needed to obtain MC proficiency is unclear. For instance, some studies report significant improvements in children’s MC after a 550 min dose over 13 weeks [53], 1,400 min dose over 8 weeks [64], 1,400 min dose over 12 months [66], and 2400 min dose over 20 weeks [75], whereas other studies fail to see significant effects after a 480 [58] or 3600-min dose [55] at five weeks and 12 months, respectively. Similarly, previous literature demonstrates inconsistencies regarding the amount of intervention needed to produce positive developmental changes in MC. Wick et al. [94], found interventions conducted from one to five-months had a larger effect on FMS than interventions lasting over six months. In addition, a recent meta-analysis study indicated that children aged 3–5 need to practice their FMS with a teacher-led intervention regularly (i.e., 3 times per week for ≤ 6 months) to achieve significant improvement in MC [95]. Specifically, Van Capelle et al. [96], suggested that interventions for increasing FMS must be implemented more than three times per week and that sessions should last longer than 30 min. However, the meta-analysis by Logan et al. [20], reported a nonsignificant relationship between effect sizes of FMS improvements and intervention duration with a dosage between 500 and 1,400 min. A possible explanation for the results is that there was heterogeneity in study length (4 to 192 weeks), frequency of program delivery (1 to 5 times per week), and duration of program sessions (15 to 120 min) across the included studies. Another possible explanation is that there may have been a “ceiling effect” in which children had already achieved better performance in the early stages of the intervention. As a result, more time (quantitative aspect) may not necessarily translate to better performance (quality aspect). Robinson et al. [93], presented that as little as 600 min of high-quality instruction during the intervention program can significantly improve children’s MC. Thus, future research is warranted. It would be beneficial to examine the impact that different intervention dosages (e.g., duration and frequency) would have on children’s MC development under similar PA intervention conditions. Additionally, most studies did not report the dose received (i.e., on-task time in the tasks/activities), which is an important area for future research because motor skill development theory shows that one of the key factors is the number of correct practice trials a child completes [97]. Ultimately, understanding patterns of change resulting from different ranges of intervention dosages could illustrate how only minimal amounts of time could lead to positive developmental changes in MC and help establish recommendations and policies for practitioners implementing CSPAPs.

Interestingly, studies involving multiple component interventions mainly addressed the FCE and SI CSPAP components [51, 54, 59, 61, 66, 69, 74, 77]. The multicomponent interventions were collaboratively delivered by a variety of facilitators, such as PE teachers, classroom teachers, administrators, coaches, community leaders, parents, and medical or healthcare staff. The results of this review parallel previous interventions that involved parents as promoters for PA and MC in their own children [98, 99]. Overall, it seems reasonable to assert that the FCE and SI components of the CSPAP framework function as important elements in the support system for PA program implementation in schools [26], and can help to enhance children’s MC. However, there is insufficient evidence specific to each component (FCE or SI) to make conclusions about the its specific contribution to MC, and further research is needed to determine which strategies are most effective for optimizing FCE and SI to support the development of MC in children.

Overall, there has been a lack of variety in theories used to guide intervention development. The studies in this review used the socio-ecological model [54, 66], motivation theory [51, 78], and social cognitive theory [55]. However, other theoretical perspectives should be considered, as well. For example, interventions could incorporate strategies such as encouraging teachers to provide positive feedback and emphasizing mastery of skills rather than competition. These practical strategies reflect constructs related to motivation theories such as Self-Determination Theory [100] and Achievement Goal Theory [101]. Moreover, there is a lack of strong process evaluation across studies. While intervention programs demonstrated improvements in children’s MC, multiple components were typically implemented simultaneously. Nearly half of the studies did not measure intervention implementation elements (e.g., fidelity and selection of participants). Additionally, some studies did not describe their instructional strategies in detail. As such, it is unclear how these variables affected study findings. It is important to show the study context and resources to improve the interpretation of research findings.

The meta-analysis results indicated that the pooled effect sizes of all interventions to increase children’s overall MC were statistically significant, with 11 studies (42%) reporting large effect sizes. However, there was a small number of heterogeneous studies included in the meta-analysis. Subsequently, a subgroup comparison between measurements was performed. Studies were separated by measurement tool (i.e., TGMD-2 or-3 vs. other measurements); the studies that used TGMD-2 or -3 had a large effect on MC [64, 75, 76], whereas other assessments had a moderate effect on MC (Hedges’ *g* = 0.79 vs. 0.57). Thus, not all PA intervention programs have the same effect on the development of MC. For example, Rudd et al. [65, 67], which assessed changes in both TGMD-2 and KTK, reported different effect sizes (Hedges’ *g* = 0.79 vs. 0.41) in the development of MC in their gymnastics intervention group. Since studies using TGMD-2 or -3 often show large effect sizes, they could thus be an effective way to assess the development of children’s MC (as opposed to other measurements). Although these measurements are commonly used for assessing children’s MC, the variety in scoring criteria protocol might provide different aspects of MC across the different movement dimensions evaluated [20, 67]. Research has also shown that there is a low-to-moderate correlation between TGMD-2 and KTK in children [102]. Therefore, further studies on the effect of PA interventions on MC should carefully consider the types of assessments (i.e., process measure or outcome of product) and their associations with intervention outcomes.

The current meta-analysis found that single PE component interventions had a larger effect on MC than other single-component approaches. A common misconception is that MC development is a naturally occurring phenomenon; however, literature suggests that it must “be practiced, taught, and reinforced through developmentally appropriate movement programs” [87]. A large number of previous studies using the CSPAP framework focus mainly on the potential of PE to provide enough amounts of PA, that is, its contribution to the achievement of daily PA recommendations [26]. The main results of the meta-analysis showed that the PE component is foundational to learning and developing MC in children [26, 103]. Previous research found that PE contributed to improving elementary school students’ manipulative skills [52] and motor skill competence [103]. In a meta-analysis study, Dudley et al. [104], also presented that PE can be efficacious in improving MC in primary school children. Moreover, the quality of instruction and time spent in practice are of utmost importance in improving MC [16]. However, limited research is devoted to studying the unique potential of PE within the CSPAP framework to develop MC in schoolchildren aged 5–12 and its impact on long-term PA trajectories [7]. Additionally, our results suggest that given the limited PE curriculum time in elementary schools, strategies to engage classroom teachers and/or parents in both school-based lessons and to support practice opportunities outside of PE class and school may be a worthwhile target for future interventions. That is, implementing a PA program using other single components in the CSPAP framework has the potential to support PE’s goal of developing children’s MC.

There was minimal effectiveness of adding other CSPAP components to PE for the development of children’s MC. Many of the interventions included in the current review were multicomponent interventions. We expected that multiple components being used to increase MC may be a more effective approach than the single PE component approach. Unfortunately, the results suggest multicomponent interventions (adding other components to PE) have had minimal impact on the development of children’s MC as indicated by the effect size values. A possible explanation for our results is that the quality of PA programs might be more important than the quantity of CSPAP components used to increase children’s MC. More research, therefore, is warranted to examine how the quality of PA experiences provided through each CSPAP component can impact the MC of school-age children.

In this review, moderation analyses were performed to explore the effectiveness of potential moderators (study length, delivery agent, and study design) on the effect size of MC with meta-regression [40]. In general, our findings suggest that the effects of potential moderator variables had no significance on children’s MC. In line with the present results, Loras [31] also showed that participants’ ages, the total amount of time for intervention, and the type of MC measurement were not statistically significant moderators of effect size. However, a handful of studies had insignificant heterogeneity, indicating substantial differences in study contexts and characteristics. Additionally, there was limited information available for some moderator variable analyses. Thus, further studies should collect and report more complete data so that potential moderators can be examined to help us better understand the effects of potential moderator variables on the development of MC.

### Strengths and limitations

To the best of our knowledge, this is the first review to examine PA intervention effects on the MC of children aged 5–12 years from a CSPAP perspective. Although previous meta-analyses on this topic have been conducted, they were limited to FMS in the early childhood age band [37, 94–96]. In this review, we included elementary school age and, not only FMS, but also motor coordination and motor proficiency, representing a broader range of MC outcomes. Additionally, our review provided insight into different intervention approaches to change children’s MC within the context of the CSPAP framework, highlighting the quality of PE as a particularly effective foundational component. Another strength of this review was that we used a sensitive search strategy to ensure relevant studies were not missed. In addition, a rigorous review methodology, including independent, duplicate reviews of selected studies, ensured most studies were captured [40].

This study also has several limitations. First, for practical reasons, we only included peer-reviewed studies published in English; non-English publications, and therefore further comparative evidence, may have been available on the topic. Second, there was noticeable heterogeneity of study approaches and assessment tools used to test children’s MC across studies, which makes it difficult to compare findings across the studies. Nevertheless, validated testing instruments were utilized across included studies which minimized a major domain of bias and further strengthened the overall evidence of this review. Third, the review included small sample sizes and some articles were feasibility studies or pilot trials. Finally, this review potentially excludes studies published after November 2021.

## Conclusions

The current review provides a unique contribution to the literature through its primary focus on considering the effectiveness of PA interventions on children’s MC from a CSPAP perspective. In light of our results, single and/or multi component intervention approaches within the CSPAP framework appear to be a promising avenue to promote MC in school-aged children (5–12 years old). This review also highlights that CSPAP-aligned PA programs should be tailored to the context within which they are delivered, most notably the PE component which can be best adapted to the context through professional teacher training. Also, combining different PA intervention strategies (e.g., goal setting and reinforcement) with the SI/FCE components should be considered to improve MC through increased engagement and motivation.

Beyond these findings, this study identified avenues for future research. To increase intervention engagement and efficacy, future studies should examine the impact of a greater emphasis on children’s MC. It is important to improve our understanding of which CSPAP-aligned PA intervention approaches are more effective than others by stratifying for the target groups, the setting, and the characteristics of the interventions. In other words, we need to identify and investigate well-designed interventions, including tailored types of PA programs. Additionally, future research should use stronger methodological approaches and consider expanding the theoretical ground for this research. Specifically, in order to make intervention studies more robust, research using high-standard randomization procedures to investigate the ability of CSPAPs to improve children’s MC is needed. Ultimately, we should pursue how to effectively translate the evidence into practice to better conceptualize CSPAPs designed for children’s MC development.

### Electronic supplementary material

Below is the link to the electronic supplementary material.

Supplementary Material File: (a) outlines the detailed search strategy and specific key terms employed to identify relevant studies focusing on outcomes related to motor skill competence in children; (b) contains figures S1-S13, offering visual representations of quality assessments, effect sizes, and publication bias assessments related to the included studies; and (c) includes a table that shows the characteristics of the included studies, specifying the population, study design, intervention focus, measurement tools, fidelity reporting, and main findings.

## Data Availability

The datasets used and/or analyzed during the current study are available from the corresponding author upon reasonable request.

## Abbreviations

CSPAP: Comprehensive School Physical Activity Program
FCE: Family and Community Engagement
FMS: Fundamental Motor Skills
MC: Motor Competence
PABAS: Physical Activity Before and After School
PADS: Physical Activity During School
PA: Physical Activity
PE: Physical Education
RCT: Randomized Controlled Trial
WHO: World Health Organization
