# Supplementary material for: Systematic review and meta-analysis of physical activity interventions to increase elementary children’s motor competence: a comprehensive school physical activity program perspective
Source: BMC Public Health. 2024 Mar 15;24:826. doi: 10.1186/s12889-024-18145-1 (PMC10943790; doi:10.1186/s12889-024-18145-1)
Supplement: Supplementary file 1 — Supplementary Material File: (a) outlines the detailed search strategy and specific key terms employed to identify relevant studies focusing on outcomes related to motor skill competence in children; (b) contains figures S1-S13, offering visual representations of quality assessments, effect sizes, and publication bias assessments related to the included studies; and (c) includes a table that shows the characteristics of the included studies, specifying the population, study design, intervention focus, measurement tools, fidelity reporting, and main findings. [file 12889_2024_18145_MOESM1_ESM.pdf]

Electronic Supplementary Material Table S1

Table S1.

Search strategy and key terms

Population	Study Design	Intervention Focus	Motor skill competence related outcomes	Database
Child*[tiab] OR Child*[mh] OR OR Boys[tiab] OR Girls[tiab] OR Student[tiab] OR Student*[tiab]	Intervention[tiab] OR Experiment[tiab] OR Program*[tiab] OR Evaluation[tiab] OR Trial[tiab] OR Random*[tiab] OR Clinic*[tiab] OR "Controlled trial"[tiab]	"Physical activity" [tiab] OR Exercise[tiab]	"Motor competence" [tiab] OR "Motor skills"[tiab] OR "Movement skill"[tiab] OR Coordination[tiab] OR "Motor development" [tiab] OR "Motor skill competence"[tiab] OR "Locomotor skills" [tiab] OR "Fundamental motor skills" [tiab] OR "Motor proficiency"[tiab] OR "Object control"[tiab]	PubMed/ Medline
Youth* or Child* or Student* or Boys or Girls or Teenager* or Teen*	Intervention or Experiment or Program or Evaluation or Trial or Random* or Clinic* or Controlled trial	Physical activity or Exercise	Motor competence or Motor skills or Movement skill or Coordination or Motor development or Motor skill competence or Locomotor skills or Fundamental motor skills or Motor proficiency or Object control	PsycINFO<br>SPORTDiscus<br>ERIC<br>CINAHL<br>Web of Science
(Youth*:ab,ti OR Child*:ab,ti OR Student*:ab,ti OR teenager*:ab,ti OR teen*:ab,ti OR Boys:ab,ti OR Girls:ab,ti) AND (Intervention:ab,ti OR Experiment:ab,ti OR Program*:ab,ti OR Evaluation:ab,ti OR Trial:ab,ti OR Random*:ab,ti OR Clinic*:ab,ti OR 'Controlled trial':ab,ti) AND ('Physical activity':ab,ti OR Exercise:ab,ti) AND ('Motor competence':ab,ti OR 'Motor skills':ab,ti OR 'Movement skill':ab,ti OR Coordination:ab,ti OR 'Motor development':ab,ti OR 'Motor skill competence':ab,ti OR 'Locomotor skills':ab,ti OR 'Fundamental motor skills':ab,ti OR 'Motor proficiency':ab,ti OR 'Object control':ab,ti)				Embase

Electronic Supplementary Material Figures S1-13

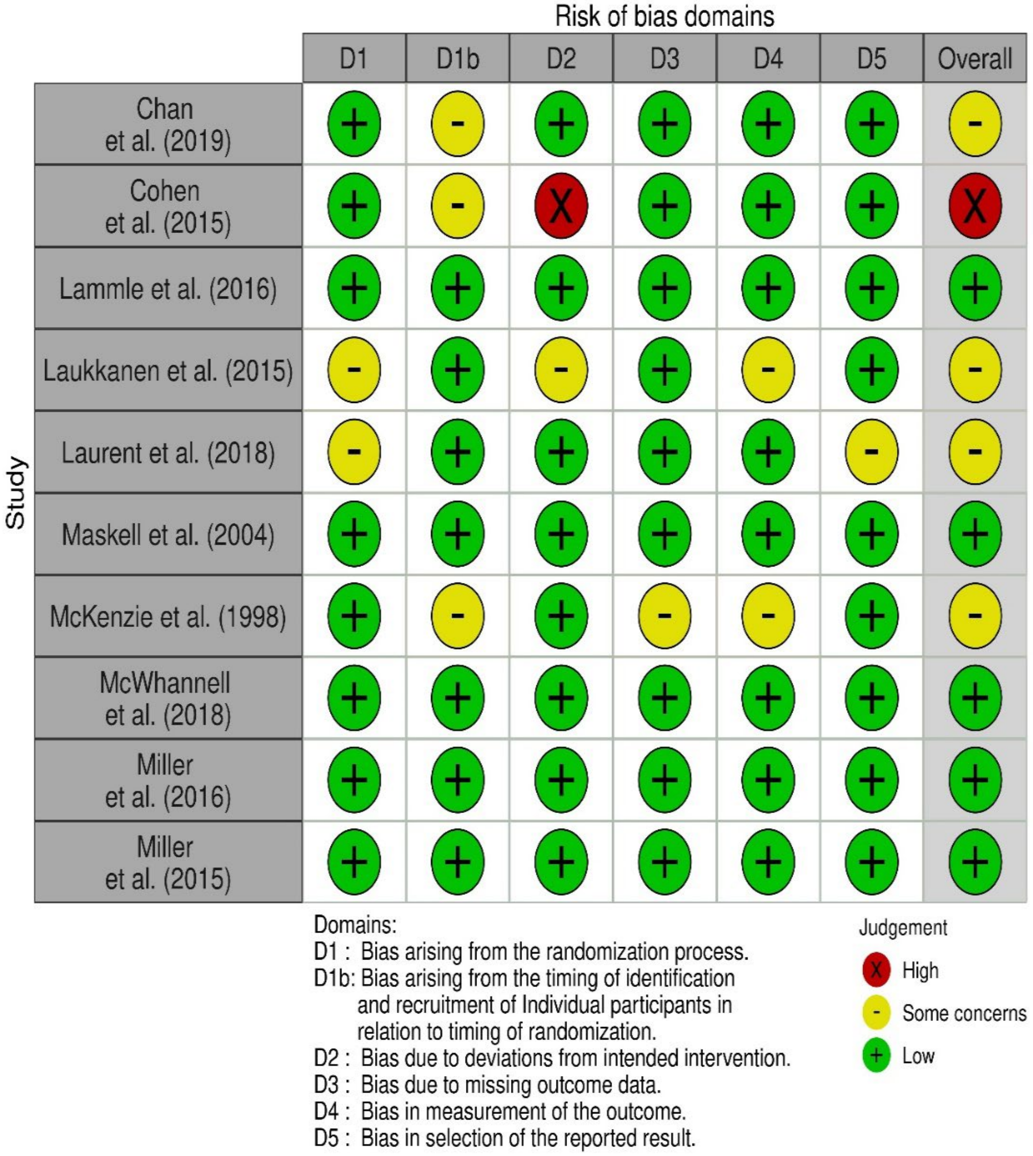

Fig S1. Quality assessment for C-RCT or RCT studies based on Revised Cochrane Risk of Bias tool for randomized trials (RoB 2.0).

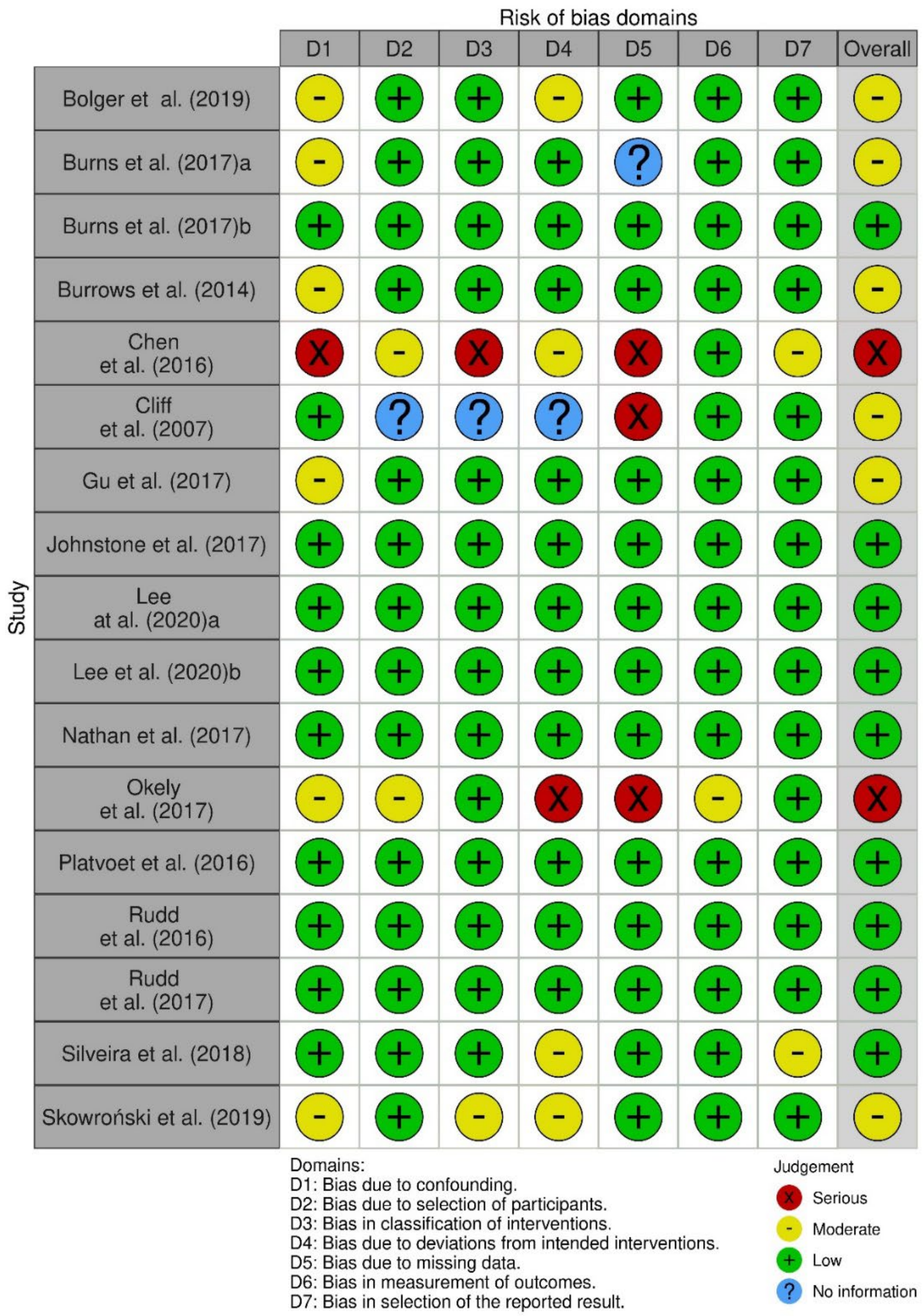

Fig S2. Quality assessment for N-RCT studies based on Risk of Bias in Non-randomized Studies of Interventions tool (ROBINS-I).

Study name	Hedges's g	Standard error	Variance	Lower limit	Upper limit	Z-Value	p-Value
	Statistics for each study						
Bolger et al. (2019)	1.929	0.177	0.031	1.583	2.276	10.923	0.000
Burn et al. (2017)a	0.421	0.027	0.001	0.367	0.474	15.419	0.000
Burn et al. (2017)b	0.595	0.035	0.001	0.527	0.664	17.005	0.000
Burrows et al. (2014)	0.349	0.358	0.128	-0.353	1.051	0.975	0.329
Chan et al. (2019)	0.685	0.124	0.015	0.442	0.927	5.530	0.000
Chen et al. (2016)	0.712	0.029	0.001	0.655	0.769	24.610	0.000
Cliff et al. (2007)	1.923	0.458	0.210	1.025	2.820	4.200	0.000
Cohen et al. (2015)	0.513	0.095	0.009	0.326	0.700	5.373	0.000
Gu et al. (2017)	1.040	0.116	0.013	0.813	1.266	8.997	0.000
Johnstone et al. (2017)	0.580	0.241	0.058	0.108	1.052	2.407	0.016
Lammle et al. (2016)	0.339	0.049	0.002	0.244	0.434	6.984	0.000
Laukkanen et al. (2015)	0.042	0.210	0.044	-0.370	0.454	0.202	0.840
Laurent et al. (2018)	1.562	0.430	0.185	0.720	2.405	3.635	0.000
Lee et al. (2020)a	1.570	0.416	0.173	0.754	2.386	3.770	0.000
Lee et al. (2020)b	0.909	0.293	0.086	0.335	1.483	3.104	0.002
Maskell et al. (2004)	0.143	0.304	0.092	-0.452	0.738	0.472	0.637
McKenzie et al. (1998)	0.647	0.091	0.008	0.468	0.825	7.107	0.000
Miller et al. (2015)	1.313	0.171	0.029	0.977	1.648	7.669	0.000
Miller et al. (2016)	0.869	0.201	0.040	0.475	1.263	4.325	0.000
Nathan et al. (2017)	0.789	0.157	0.025	0.482	1.097	5.029	0.000
Okely et al. (2017)	0.588	0.074	0.005	0.443	0.733	7.948	0.000
Platvoet et al. (2016)	0.427	0.106	0.011	0.219	0.634	4.030	0.000
Rudd et al. (2016)	0.480	0.118	0.014	0.250	0.710	4.083	0.000
Rudd et al. (2017)	0.437	0.189	0.036	0.067	0.808	2.312	0.021
Silveria et al. (2018)	0.442	0.335	0.112	-0.214	1.098	1.321	0.187
Skowroski et al. (2019)	1.485	0.411	0.169	0.679	2.291	3.610	0.000
	0.705	0.056	0.003	0.595	0.814	12.628	0.000

Hedges's g and 95% CI

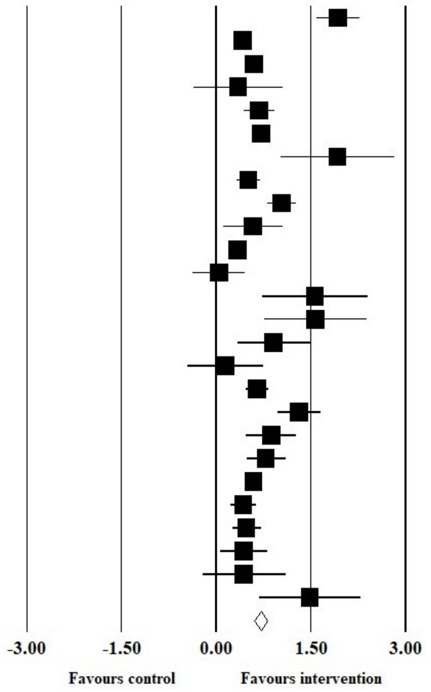

Fig S3. Forest plot for pooled effect sizes (Hedges' g) of all CSPAP-aligned PA intervention on children's MC from random effects meta-analysis of eligible studies.

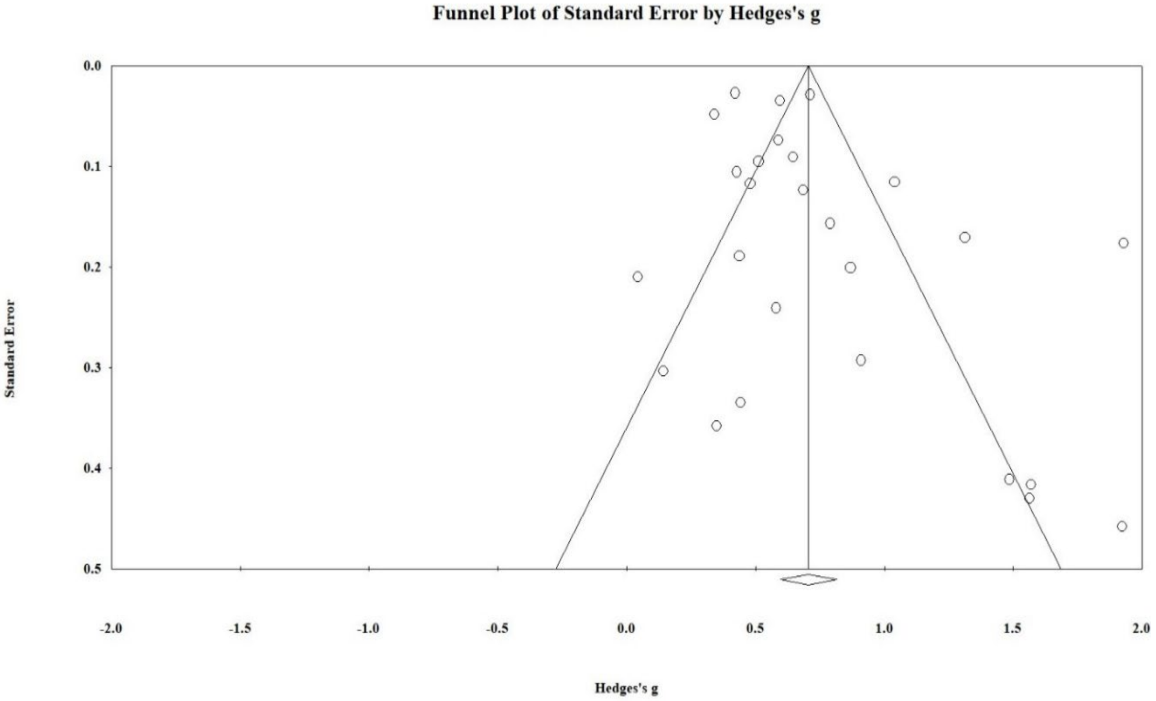

Fig S4. Funnel plot for assessment of publication bias using pooled analysis of effect sizes of MC outcomes.

Study name	Hedges's g	Standard error	Variance	Lower limit	Upper limit	Z-Value	p-Value
Bolger et al. (2019)	1.929	0.177	0.031	1.583	2.276	10.923	0.000
Burn et al. (2017)a	0.421	0.027	0.001	0.367	0.474	15.419	0.000
Burn et al. (2017)b	0.595	0.035	0.001	0.527	0.664	17.005	0.000
Burrows et al. (2014)	0.349	0.358	0.128	-0.353	1.051	0.975	0.329
Chan et al. (2019)	0.685	0.124	0.015	0.442	0.927	5.530	0.000
Cliff et al. (2007)	1.923	0.458	0.210	1.025	2.820	4.200	0.000
Cohen et al. (2015)	0.513	0.095	0.009	0.326	0.700	5.373	0.000
Johnstone et al. (2017)	0.580	0.241	0.058	0.108	1.052	2.407	0.016
Lee et al. (2020)a	1.570	0.416	0.173	0.754	2.386	3.770	0.000
Lee et al. (2020)b	0.909	0.293	0.086	0.335	1.483	3.104	0.002
Maskell et al. (2004)	0.143	0.304	0.092	-0.452	0.738	0.472	0.637
Miller et al. (2015)	1.313	0.171	0.029	0.977	1.648	7.669	0.000
Miller et al. (2016)	0.869	0.201	0.040	0.475	1.263	4.325	0.000
Nathan et al. (2017)	0.789	0.157	0.025	0.482	1.097	5.029	0.000
Rudd et al. (2016) TGMD	0.534	0.118	0.014	0.303	0.765	4.527	0.000
Rudd et al. (2017) TGMD	0.247	0.188	0.035	-0.121	0.615	1.317	0.188
Skowroski et al. (2019)	1.485	0.411	0.169	0.679	2.291	3.610	0.000
	0.787	0.082	0.007	0.626	0.948	9.570	0.000

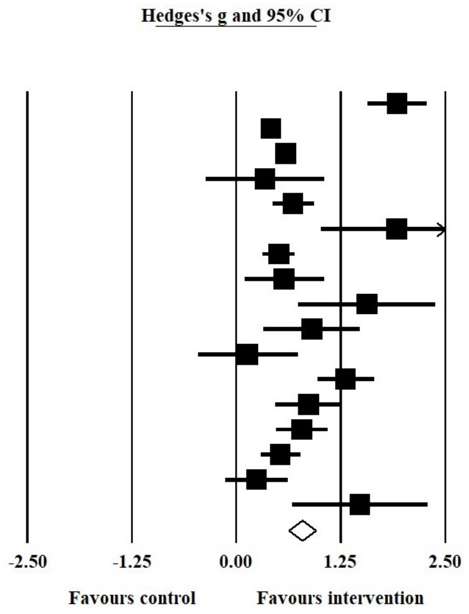

Fig S5. Forest plot for pooled effect sizes (Hedges' g) of all CSPAP-aligned PA interventions using the TGMD-2 or -3 tool on children's MC from random effects meta-analysis of eligible studies.

Study name	Hedges's g	Standard error	Variance	Lower limit	Upper limit	Z-Value	p-Value
	Statistics for each study						
Chen et al. (2016)	0.712	0.029	0.001	0.655	0.769	24.610	0.000
Gu et al. (2017)	1.040	0.116	0.013	0.813	1.266	8.997	0.000
Lammle et al. (2016)	0.339	0.049	0.002	0.244	0.434	6.984	0.000
Laukkanen et al. (2015)	0.042	0.210	0.044	-0.370	0.454	0.202	0.840
Laurent et al. (2018)	1.562	0.430	0.185	0.720	2.405	3.635	0.000
McKenzie et al. (1998)	0.647	0.091	0.008	0.468	0.825	7.107	0.000
Okely et al. (2017)	0.588	0.074	0.005	0.443	0.733	7.948	0.000
Platvoet et al. (2016)	0.427	0.106	0.011	0.219	0.634	4.030	0.000
Rudd et al. (2016) KTK	0.449	0.117	0.014	0.219	0.679	3.828	0.000
Rudd et al. (2017) KTK	0.510	0.190	0.036	0.138	0.882	2.685	0.007
Silveria et al. (2018)	0.442	0.335	0.112	-0.214	1.098	1.321	0.187
	0.572	0.077	0.006	0.421	0.722	7.448	0.000

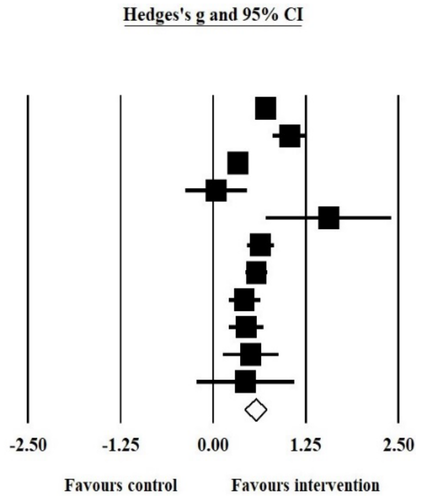

Fig S6. Forest plot for pooled effect sizes (Hedges' g) of all CSPAP-aligned PA interventions using other measurement tools on children's MC from random effects meta-analysis of eligible studies.

Study name	Hedges's g	Standard error	Variance	Lower limit	Upper limit	Z-Value	p-Value
Laukkanen et al. (2015)	0.042	0.210	0.044	-0.370	0.454	0.202	0.840
Platvoet et al. (2016)	0.427	0.106	0.011	0.219	0.634	4.030	0.000
Rudd et al. (2016)	0.449	0.117	0.014	0.219	0.679	3.828	0.000
Rudd et al. (2017)	0.510	0.190	0.036	0.138	0.882	2.685	0.007
Silveria et al. (2018)	0.442	0.335	0.112	-0.214	1.098	1.321	0.187
	0.406	0.067	0.005	0.274	0.538	6.034	0.000

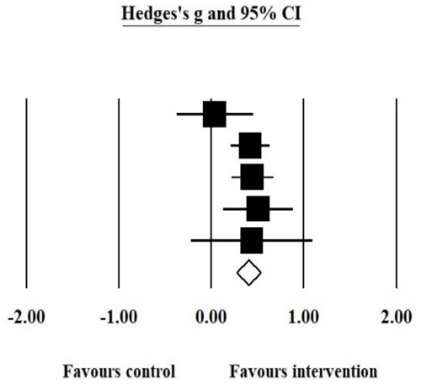

Fig S7. Forest plot for pooled effect sizes (Hedges' g) of all CSPAP-aligned PA interventions using KTK tool on children's MC from random effects meta-analysis of eligible studies.

Study name	Hedges's g	Upper limit	Standard error	Variance	Lower limit	Z-Value	p-Value
	Statistics for each study						
Bolger et al. (2019)	1.929	2.276	0.177	0.031	1.583	10.923	0.000
Chan et al. (2019)	0.685	0.927	0.124	0.015	0.442	5.530	0.000
Chen et al. (2016)	0.712	0.769	0.029	0.001	0.655	24.610	0.000
Gu et al. (2017)	1.040	1.266	0.116	0.013	0.813	8.997	0.000
Maskell et al. (2004)	0.143	0.738	0.304	0.092	-0.452	0.472	0.637
Miller et al. (2015)	1.313	1.648	0.171	0.029	0.977	7.669	0.000
Platvoet et al. (2016)	0.427	0.634	0.106	0.011	0.219	4.030	0.000
Rudd et al. (2016)	0.534	0.765	0.118	0.014	0.303	4.527	0.000
Rudd et al. (2017)	0.247	0.615	0.188	0.035	-0.121	1.317	0.188
	0.794	1.036	0.123	0.015	0.553	6.442	0.000

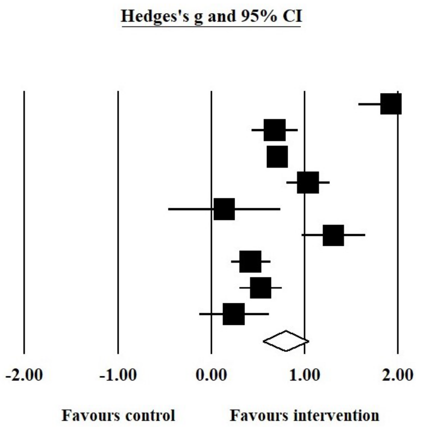

Fig S8. Forest plot for pooled effect sizes (Hedges' g) of PE single component interventions on children's total MC from random effects meta-analysis of eligible studies.

Study name	Hedges's g	Standard error	Variance	Lower limit	Upper limit	Z-Value	p-Value
	Statistics for each study						
Burrows et al. (2014)	0.349	0.358	0.128	-0.353	1.051	0.975	0.329
Johnstone et al. (2017)	0.603	0.241	0.058	0.130	1.075	2.498	0.012
Lammle et al. (2016)	0.339	0.049	0.002	0.244	0.434	6.984	0.000
Laukkanen et al. (2015)	0.042	0.210	0.044	-0.370	0.454	0.202	0.840
Laurent et al. (2018)	1.562	0.430	0.185	0.720	2.405	3.635	0.000
Lee et al. (2020)a	0.833	0.381	0.145	0.087	1.579	2.189	0.029
Lee et al. (2020)b	0.421	0.282	0.079	-0.131	0.973	1.495	0.135
Okely et al. (2017)	0.588	0.074	0.005	0.443	0.733	7.948	0.000
	0.483	0.101	0.010	0.286	0.681	4.796	0.000

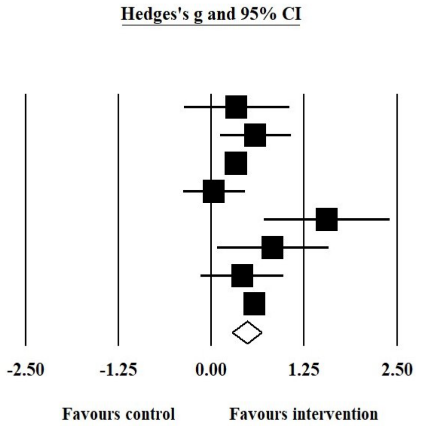

Fig S9. Forest plot for pooled effect sizes (Hedges' g) of non-PE single component interventions on children's total MC from random effects meta-analysis of eligible studies.

Study name	Statistics for each study						
	Hedges's g	Standard error	Variance	Lower limit	Z-Value	Upper limit	p-Value
Lammle et al. (2016)	0.339	0.049	0.002	0.244	6.984	0.434	0.000
Okely et al. (2017)	0.588	0.074	0.005	0.443	7.948	0.733	0.000
Johnstone et al. (2017)	0.603	0.241	0.058	0.130	2.498	1.075	0.012
	0.477	0.107	0.012	0.267	4.443	0.688	0.000

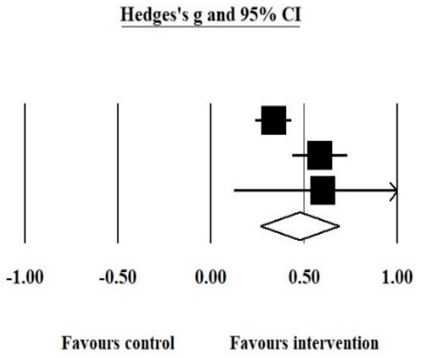

Fig S10. Forest plot for pooled effect sizes (Hedges' g) of PADS single component interventions on children's total MC from random effects meta-analysis of eligible studies.

Study name	Hedges's g	Standard error	Variance	Lower limit	Z-Value	Upper limit	p-Value
Burrows et al. (2014)	0.349	0.358	0.128	-0.353	0.975	1.051	0.329
Lee et al. (2020) Need	0.421	0.282	0.079	-0.131	1.495	0.973	0.135
Lee et al. (2020) FMS	0.833	0.381	0.145	0.087	2.189	1.579	0.029
	0.505	0.191	0.037	0.130	2.638	0.880	0.008

Statistics for each study

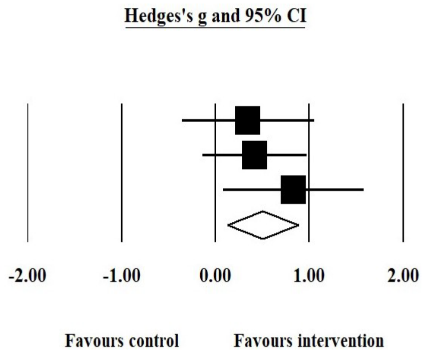

Fig S11. Forest plot for pooled effect sizes (Hedges' g) of PABAS single component interventions on children's total MC from random effects meta-analysis of eligible studies.

Study name	Hedges's g	Upper limit	Standard error	Variance	Lower limit	Z-Value	p-Value
McKenzie et al. (1998)	0.463	0.639	0.090	0.008	0.287	5.151	0.000
Miller et al. (2016)	0.869	1.263	0.201	0.040	0.475	4.325	0.000
Skowroski et al. (2019)	0.786	1.529	0.379	0.144	0.043	2.074	0.038
	0.634	0.936	0.154	0.024	0.332	4.115	0.000

(Statistics for each study)

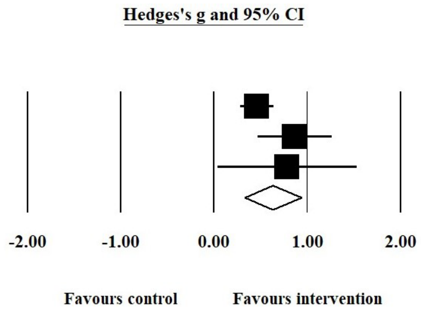

Fig S12. Forest plot for pooled effect sizes (Hedges' g) of PE and one additional CSPAP component (PE+1) interventions on children's total MC from random effects meta-analysis of eligible studies.

Study name	Hedges's g	Upper limit	Standard error	Variance	Lower limit	Z-Value	p-Value
	**Statistics for each study**						
Burn et al. (2017) Effect	0.421	0.474	0.027	0.001	0.367	15.419	0.000
Nathan et al. (2017)	0.789	1.097	0.157	0.025	0.482	5.029	0.000
Silveria et al. (2018)	0.442	1.098	0.335	0.112	-0.214	1.321	0.187
	0.543	0.816	0.139	0.019	0.271	3.908	0.000

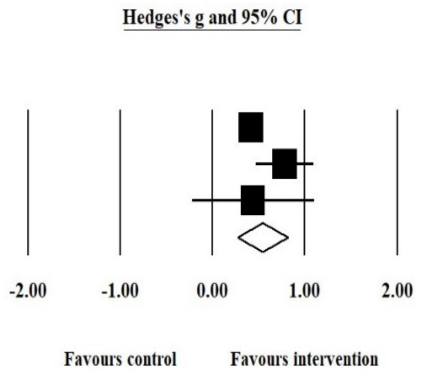

Fig S13. Forest plot for pooled effect sizes (Hedges' g) of PE and two additional CSPAP component (PE+2) interventions on children's total MC from random effects meta-analysis of eligible studies.

Electronic Supplementary Material Appendix S1

Appendix S1.

Characteristics of included studies examining MC

Study	Study design	CSPAP component	Population, Sample size, Sex, and Age	Duration & Dose	Description of PA intervention	Intervention deliverer	Measurement of MC	Fidelity	Main findings in MC Outcomes as reported
Bolger et al. (2019).	QE	PE	*N* = 357 (195 INT; 162 CONT) from 2 INT classes and 1 control classes in 1 school in 2 urban and 1 rural in Ireland.<br><br>Sex F/M (*n*): 173/184<br><br>Age: 6-11 years	Total duration: 26 weeks<br><br>Frequency: 2 times per week<br><br>Length: 25 minutes	Project Energize program: a primary school-based PA, health, and nutrition INT program.<br><br>Each of the PE lessons incorporated the revision of four FMS activities that accounted for cultural, environmental and curriculum differences.	PE teachers	TGMD-2	Measure: Not reported<br><br>Delivered as intended: Not reported	The INT group's locomotor standard score, object-control standard score, and GMQ significantly improved from pre- to post-INT compared to the CONT group, which significantly dis-improved.<br><br>A group-time interaction effect was found in favor of the INT group for locomotor standard score, object-control standard score, and GMQ.
Burns et al. (2017a)	QE	PE, PADS, SI	*N* = 1,460 from 3 schools in USA.<br><br>Sex F/M (*n*): 730/730<br><br>Age: 8.4 ± 1.8 years	Total duration: 12 weeks<br><br>Frequency: one, plus recess and classroom PA (once a week required suggested 3 times a week)<br><br>Length: 50 minutes for PE, 10 for	CSPAP on gross motor skills development: Dynamic Physical Education for Elementary School Children curriculum during PE lessons, PA engagement opportunities throughout the school day during recess and regular classroom time (during which teachers integrated PA into academic lessons and classroom activity breaks via stretching, walking, jumping, or relaxation activities), and SI that provided teacher	PE teachers and classroom teachers	TGMD-2	Measure: Observations (field notes) and teacher reports<br><br>Delivered as intended: High	The INT students' MC percent scores improved at the post-test compared with the baseline ($p < .001$).

				classroom PA minutes	professional training to increase the quality of PE.				
Burns et al. (2017b)	QE	PE, PADS, PABAS, SI	*N* = 976 from 5 schools in Salt Lake City district in USA.<br><br>Sex F/M (*n*): 413/563<br><br>Age: 9.1 ± 1.5 years	Total duration: 36 weeks<br><br>Frequency: PE= 1 day, Recess 2 a day (10 a week), MI= at least one per day<br><br>Length: PE= 50 min; Recess=15 min; MI= 10 min	CSPAP program: Aimed to provide children PA opportunities throughout the school day during specific leisure times (i.e., recess) and integrated PA that focused on motor skill development into academic lessons and classroom activities with continuous teacher training and assistance.	PE teachers, physical activity leaders, and classroom teachers	TGMD-3	Measure: confirmed by interviews and spot checks by research team<br><br>Delivered as intended: High	There were statistically significant coefficients for time ($p < .001$) and an age × time interaction ($p < .001$) on children's MC. Significant improvements were reported for locomotor and object control skills ($p < .001$).
Burrows et al. (2014)	QE	PABAS	*N* = 40 (25 games-program group, 15 sports-program group) in rural area in Canada<br><br>Sex F/M (*n*): 16/9 (games), 7/8 (sports)<br><br>Age: 7.87 ± 1.07 years (games)<br><br>8.37 ± 1.27 years (sports)	Total duration: 11 weeks<br><br>Frequency: 5 times per week<br><br>Length: 60 minutes	Two after school programs: A low-organized games (outside) and sports-based program (indoors) model including swimming activities, floor hockey, and soccer activities.	After school program leaders	TGMD-2	Measure: Not reported<br><br>Delivered as intended: Not reported	The sports-based program participants showed no improvement in FMS ($p = .91$), and the games-based program participants significantly improved their proficiency ($p < .05$).
Chan et al. (2019)	C-RCT	PE	*N* = 282 from 5 INT classes and 5 CONT classes in 5 schools	Total duration: 13 weeks (a total 550 minutes)	Assessment for learning intervention program: Fun movement activities related to specific motor skills were implemented and aligned with the	PE teachers	TGMD-3	Measure: Observations of program delivery using	The INT group's locomotor skills and overall FMS competence from baseline to post-intervention were greater ($p < .001$) than the CONT group.

			in urban in China<br><br>Sex F/M (*n*): 194/84<br><br>Age: 8.4 ± 0.56 years	Frequency: 1-2 times per week<br><br>Length: 45-70 minutes	evaluation criteria of TGMD-3 to facilitate formative assessment.			checklist, lesson plans<br><br>Delivered as intended: High	There was no significant difference between the groups for object control skills (p = .116) .
Chen et al. (2016)	QE	PE	*N* = 4,234 (1377 soccer; 1496 overhand; 1361 forehand) from 9 schools in suburban in USA<br><br>Sex F/M (*n*): 2,032/2,202<br><br>Age: 6-11 years	Total duration: 2 years<br><br>Frequency: 1 time per week<br><br>Length: 60 minutes	CATCH PE Curriculum: designed as a comprehensive elementary school PE and nutrition program, which includes motor skill themes and physical fitness activities.	PE teachers	PE Metrics	Measure: Video recorded assessed using Quality of Teaching Rubrics, lesson plans<br><br>Delivered as intended: High	The CATCH PE was conducive to improving fourth- and fifth-grade students' motor skill competency in the three FMS skills.
Cliff et al. (2007)	QE	PABAS, FCE	*N*= 13 in Australia<br><br>Sex F/M (*n*): 8/5<br><br>Age: 10.4 ± 1.2 years	Total duration: 10 weeks<br><br>Frequency: 1 time per week<br><br>Length: 120 minutes	SHARK program: A community-based program with an additional home-based PA motor development program. Family participation and parental support along with skill practice outside the program (home tasks/activities). Researched approach centered around improving FMS and social support in children to increase their PA, self-esteem, and enjoyment.	Researcher and parents	KTK	Measure: Not reported<br><br>Delivered as intended: Not reported	There were significant improvements from pre- to post-test in the intervention (p < .001).
Cohen et al. (2015)	C-RCT	PE, PADS, SI, FCE	*N* = 460 (199 INT; 261 CONT) from 25 classes in 8 schools in Australia	Total duration: 48 weeks<br><br>Frequency: 1 time per week<br><br>Length:	SCORES program: A multicomponent PA and FMS intervention program through teacher professional learning, and student leadership workshops	Teachers, principals, parents, and community leaders	TGMD-2	Measure: Observation checklist<br><br>Delivered as intended: High	There was a statistically significant group–time interaction for overall FMS, with children in the INT group scoring significantly higher (p <.05) than those in the CONT group.

			Sex F/M (*n*):<br>173/184<br><br>Age:<br>8.5 ± 0.7 years	120 minutes	Used a range of approaches targeting the home environment (newsletters, parent evening, and FMS homework) to engage parents and encourage them to support their children's MC.				
Gu et al. (2017)	QE	PE	*N* = 273<br>(200 INT;<br>73 CONT)<br>from 3 schools in USA<br><br>Sex F/M (*n*):<br>137/136<br><br>Age:<br>10.89 ± 0.80 years	Total duration:<br>8 weeks<br><br>Frequency:<br>3 times per week<br><br>Length:<br>45 minutes	Goal setting and Pedometer-based PA INT program:<br>A goal-setting strategy throughout the INT can stimulate elementary children's adaptive motivation during PE by encouraging children to reach their goals monitored by pedometers.	PE teachers	PE Metrics	Measure:<br>Not reported<br><br>Delivered as intended:<br>Not reported	The INT group had significantly higher MC compared to the CONT group ($p < .001$).
Johnstone et al. (2017)	QE	PADS	*N* = 336<br>(291 INT; 45 CONT) from 11 classes in 7 schools in UK<br><br>Sex F/M (*n*):<br>152/139 (INT group),<br>30/15 (CONT group)<br><br>Age:<br>7 ± 1.1 years (INT),<br>7.4 ± 0.9 years (CONT)	Total duration:<br>20 weeks<br><br>Frequency:<br>2 times per week<br><br>Length:<br>60 minutes	Go2Play Active Play:<br>The program combined structured games and free play to increase children's FMS.	Research team	TGMD-2	Measure:<br>Not reported<br><br>Delivered as intended:<br>Not reported	The INT group had significant interaction for GMQ score ($p < .001$) and percentile ($p < 0.05$), locomotor skills score and percentile (both $p = 0.02$) but no significant interaction for object control skills score ($p = 0.1$) and percentile ($p = 0.3$) at pre- and post- assessment.
Lammle et al. (2016)	CRCT	PADS	*N* = 1,736<br>(957 INT; 779 CONT) from 81 INT classes and 76 CONT classes	Total duration:<br>1 school year<br><br>Frequency:<br>daily from 13 PA units	Join the Healthy Boat:<br>A school-based, teacher-centered health promotion program.<br><br>The INT program includes 13 PA teaching units and short daily exercises in	PE teachers and trained research Staff	DKT	Measure:<br>Not reported<br><br>Delivered as intended:<br>Not reported	The INT group showed significant improvement in the motor skills ($p < .05$) in comparison to the CONT group.

			in 91 schools in Germany<br><br>Sex F/M (*n*): 866/870<br><br>Age: 7.1 ± 0.6 years	Length: 10-15 minutes	class to increase PA, which work to target an increase in children's FMS.				
Laurent et al. (2018)	CRCT	PABAS, FCE	*N* = 28 (17 INT; 11 CONT) from 1 school in USA<br><br>Sex F/M (*n*): 15/13<br><br>Age: 9.3 ± 1.5 years	Total duration: 6 weeks<br><br>Frequency: 2 times per week<br><br>Length: 60 minutes	Resistance-training program, movement programs from university or off-campus training facility.	Coaches and research team	Functional Movement	Measure: Observation checklist<br><br>Delivered as intended: High	The INT group achieved greater improvements in FMS score ($p < .001$), relative to CONT group.
Laukkanen et al. (2015)	RCT	FCE	*N* = 89 (44 INT; 45 CONT) from 2 INT classes and 1 CONT classes in 1 school in Finland<br><br>Sex F/M (*n*): 173/184<br><br>Age: 6.16 ± 1.13 years	Total duration: 48 weeks<br><br>Frequency: N/A<br><br>Length: N/A	The PA INT program took place at a university or other off-school site (community-based).<br><br>Family involvement and counseling parents lead to an increase in children's MC.	Parents and research team	KTK and TCB	Measure: Observation checklist<br><br>Delivered as intended: High	The INT group's mean score of KTK ($p < .001$) increased significantly with time, not TCB.
Lee at al. (2020a)	QE	PABAS	*N* = 31 (20 INT; 11 CONT) from 1 school in USA<br><br>Sex F/M (*n*): 18/13<br><br>Age: 6.65 ± 0.91 years	Total duration: 8 weeks<br><br>Frequency: 3 times per week<br><br>Length: 60 minutes	The FMS INT program aimed to promote FMS competence by focusing on the mastery of 12 basic motor skills.	Research team	TGMD-2	Measure: Field observations<br><br>Delivered as intended: High	The INT group who participated in the FMS-based afterschool program showed significant improvements in FMS competence ($p < .001$), compared to the CONT group.

Lee et al. (2020b)	QE	PABAS	*N* = 35<br>(24 INT; 11 CONT) from 1 INT classes and 2 CONT classes in 3 schools in USA<br><br>Sex F/M (*n*): 22/13<br><br>Age: 6.52 ± 0.97 years	Total duration: 8 weeks<br><br>Frequency: 3 times per week<br><br>Length: 60 minutes	INT PA program aimed at teaching the 12 basic motor kills from the TGMD-2 criteria.<br>CONT group program included unsupervised free play and academic tutoring with no motor skill related instructions.	Research team	TGMD-2	Measure: Not reported<br><br>Delivered as intended: Not reported	There were significant group differences between the INT and CONT group in FMS competence ($p < .001$), non-significant gender differences between boys and girls in FMS competence ($p = .85$), and non-significant interaction effects over time ($p = .52$).<br>The effect size reported significant improvements on the INT group's FMS competence, with a medium to large effect size (0.49–1.92), in comparison to the CONT group.
Maskell et al. (2004)	CRCT	PE	*N* = 42<br>(20 INT; 22 CONT) from 1 INT class and 1 CONT class in 1 school in urban in USA<br><br>Sex F/M (*n*): 23/19<br><br>Age: 6.98 ± 0.42 years	Total duration: 5 weeks<br><br>Frequency: N/A (total 16 lessons)<br><br>Length: 30 minutes	Brain Gym movement program:<br>Designed to engage students in moving different body parts across the body midline. The INT program is a series of simple-challenging fundamental movement skills intended to enhance children's cognitive processing, psychomotor and whole-brain learning.	Research team	TGMD-2	Measure: Not reported<br><br>Delivered as intended: Not reported	There were no significant pre- or post- test group differences in TGMD-2 scores ($p = .26$).
McKenzie et al. (1998)	CRCT	PE, SI	*N* = 467<br>(201 INT; 266 CONT) from 56 classes in 7 schools in suburban area in USA<br><br>Sex F/M (*n*): 109/358<br><br>Age: N/A	Total duration: 2 school years<br><br>Frequency: 3 times per week<br><br>Length: 30 minutes	SPARK PE curriculum:<br>A comprehensive program designed to enhance both children's health related physical fitness and sports/motor skills as they participated in high levels of enjoyable PA through a classroom teacher professional development program.	Trained PE teachers and classroom teachers	N/A	Measure: Not reported<br><br>Delivered as intended: Not reported	The INT group reported the most improvement in total motor skills—gain scores were significant for catching ($p < .05$) and throwing ($p < .05$). In contrast, INT effects did not differ by gender or grade.
McWhann ell	CRCT	PABAS	*N* = 146 from 16 schools	Total duration:	A-CLASS Project:	Trained coaches	N/A	Measure: Not reported	Boys in the INT group were significantly more proficient than

et al.<br>(2018)			in Northern Ireland<br><br>Sex F/M (*n*):<br>89/57<br><br>Age:<br>9.6 ± 0.3 years	26 weeks<br><br>Frequency:<br>2 times per week<br><br>Length:<br>60 minutes	Aimed to quantify the effectiveness of structured and unstructured PA programs<br>on children's PA, FMS, physical self-perception, and self-esteem.			Delivered as intended:<br>Not reported	girls, being more improved in seven out of the eight FMSs; however these differences were only significant ($p < .01$) in the four object control skills (catch, overarm throw, strike, and kick).
Miller<br>et al.<br>(2016)	RCT	PE,<br>SI	$N = 107$<br>(55 INT;<br>52 CONT)<br>from 4 classes<br>in 1 school<br>in Australia<br><br>Sex F/M (*n*):<br>48/59<br><br>Age:<br>10.87 ± 0.87 years	Total duration:<br>6 weeks<br><br>Frequency:<br>1 time per week<br><br>Length:<br>60 minutes	PLUNGE INT program:<br>PE lessons based on game-centered curriculum;<br>Aimed to improve the children's FMS, game skills, in-class MVPA and enjoyment of PA through a professional learning process involving classroom teacher education and mentoring.	Classroom teachers	TGMD-2	Measure:<br>lesson observations<br><br>Delivered as intended:<br>High	The INT group's treatment effect significantly increased in FMS (throw and catch; $p < .001$).<br>The treatment effect significantly increased the INT group's FMS ($p < .001$).
Miller<br>et al.<br>(2015)	CRCT	PE	$N = 168$<br>(97 INT;<br>71 CONT)<br>from 6 classes<br>in 7 schools<br>in Australia<br><br>Sex F/M (*n*):<br>110/58<br><br>Age:<br>11.2 ± 1.00 years	Total duration:<br>7 weeks<br><br>Frequency:<br>N/A<br><br>Length:<br>N/A	PLUNGE program:<br>Aimed to increase the complexity of challenges experienced through gameplay-situated learning for the improvement of FMS.	Researchers and teachers	TGMD-2	Measure:<br>observations<br><br>Delivered as intended:<br>High	The treatment effect significantly increased the INT group's FMS ($p < .001$).
Nathan et al. (2017)	QE	PE,<br>PADS,<br>SI	$N = 174$<br>(83 INT; 91 CONT)<br>from 2 schools<br>in Australia<br><br>Sex F/M (*n*):<br>89/85<br><br>Age:<br>6.1 ± 0.9 years	Total duration:<br>10 weeks<br><br>Frequency:<br>2 times per week<br><br>Length:<br>30 minutes	The GLASS PA INT program:<br>Included trained students who instructed their peers to improve FMS during PE lessons and classroom settings, and trained teachers supporting their peers' instruction, which contributed an SI component to the program.	Classroom teachers and peer leaders (students)	TGMD-3	Measure:<br>checklist, observation<br><br>Delivered as intended:<br>Not reported	The treatment effect on the INT group's overall object control skills was statistically significant<br>($p < .001$).

Okely et al. (2017)	QE	PADS	*N* = 407 (223 INT; 184 CONT) from 8 schools in Australia<br><br>Sex F/M (*n*): 307/100<br><br>Age: 8.69 ± 1.7 years	Total duration: 44 months<br><br>Frequency: 2 times per week<br><br>Length: 25 minutes	PALDC INT program: A whole-of-school health promotion approach; Aimed to develop children's FMS with a focus on initiating sustainable changes in the delivery of FMS in a school context by modifying the physical and social environment and developing links with the home and local community.	Classroom teachers	N/A	Measure: Not reported<br><br>Delivered as intended: Not reported	There was a significantly greater increase in the INT group's total FMS ($p < .01$), compared with the CONT group.
Platvoet et al. (2016)	QE	PE	*N* = 425 (294 INT; 131 CONT) from 6 schools in Netherlands<br><br>Sex F/M (*n*): 233/192<br><br>Age: 6.4 ± 0.52 years	Total duration: 4 weeks<br><br>Frequency: 2 times per week<br><br>Length: 45 minutes	A goal-directed learning INT program: Aimed to improve FMS performance based on information in the Dutch national handbook for PE in primary education; PE teachers stimulated goal-directed learning, skill-specific exercises, and individual practice in PE lessons.	PE teachers	KTK	Measure: Not reported<br><br>Delivered as intended: Not reported	The INT group improved their FMS performance significantly more than the CONT group ($p < .001$).
Rudd et al. (2016)	QE	PE	*N* = 333 (135 INT; 198 CONT) from 14 INT classes and 6 CONT classes in 3 schools in Australia<br><br>Sex F/M (*n*): 169/164<br><br>Age: 8.1±1.1 years	Total duration: 16 weeks<br><br>Frequency: 1 time per week<br><br>Length: 120 minutes	Gymnastics curriculum developed by Gymnastics Australia: Aimed to develop stability, locomotive and object control skills, and general body coordination.	Gymnastics coaches, classroom teachers, and PE teachers	TGMD-2 and KTK	Measure: Lesson observations<br><br>Delivered as intended: High	The INT group showed a significant improvement compared to the CONT group in stability and object control ($p < .05$) but not in locomotor skills ($p > .05$).
Rudd et al. (2017)	QE	PE	*N* = 113 (56 INT; 57 CONT) from 2 classes in 1 school	Total duration: 8 weeks<br><br>Frequency:	LaunchPad program: Gymnastics curriculum aimed to develop children's MC.	PE teachers	KTK and TGMD-2	Measure: Not reported<br><br>Delivered as intended: Not reported	The INT group showed a significant improvement ($p < .001$), compared to the CONT group in FMS.

			in urban area in Australia<br><br>Sex F/M (*n*): 52/61<br><br>Age: 9.4±1.8 years	2 times per week<br><br>Length: 60 minutes					
Silveira et al. (2018)	QE	PE, FCE, SI	*N* = 357 (17 INT; 18 CONT) from 2 INT classes and 1 CONT classes in 1 school in Brazil<br><br>Sex F/M (*n*): 173/184<br><br>Age: 7-12 years	Total duration: 12 weeks<br><br>Frequency: 2 times per week<br><br>Length: 60 minutes	ACTION FOR HEALTH program: A multicomponent INT program included physical exercise sessions during PE lessons (e.g., circuit training, aerobic/sports activities, and recreational games), parent support to promote PA during after school classes, and nutritional education sessions (e.g., goal setting and dietary counselling with parents).	PE teachers, medical or healthcare staff, parents, and research team	KTK	Measure: Not reported<br><br>Delivered as intended: Not reported	The INT group demonstrated significant interaction (group*time) improvements ($p < .001$).
Skowrońsk i et al. (2019)	QE	PE, PABAS	*N* = 31 (20 INT; 11 CONT) from 2 classes in 2 schools in urban area in Poland<br><br>Sex F/M (*n*): 16/15<br><br>Age: 7.19 ± 0.28 years	Total duration: 1 school year (1440 min total)<br><br>Frequency: 4 times per week<br><br>Length: 45 minutes	From Fun to Sport program: PE lessons combined with an extracurricular after school program with an emphasis on the development of children's FMS	PE teachers	TGMD-2	Measure: Not reported<br><br>Delivered as intended: Not reported	There were statistically significant differences in the level of FMS between the INT group and CONT group at post-assessment ($p < .05$).

Notes: A-CLASS=Active City of Liverpool, Active Schools and SportsLinx; CATCH=Child and Adolescent Trial for Cardiovascular Health; CONT=Control; CRCT=Cluster Randomized Control Trial; DTK=Dordel Kock Test; FCE=Family and Community Engagement; FMS=Fundamental Motor Skill; GLASS=Great Leaders Active StudentS; GMQ=Gross Motor Quotient; INT=Intervention; KTK=Körperkoordinationstest Für Kinder; MC=Motor Competence; MI= Movement Integration; MVPA=Moderate to Vigorous Physical Activity; PA=Physical Activity; PABAS=Physical Activity Before and After School; PADS=Physical Activity During School; PALDC=Physical Activity in Linguistically Diverse Communities; PE=Physical Education; PLUNGE=Professional Learning for

Understanding Games Education; QE=Quasi Experimental; RCT= Randomized Control Trial; SCORES=Supportive Children's Outcome using Rewards, Exercise, and Skills; SI=Staff Involvement; SPARK=Sports, Play and Active Recreation for Kids; TCB=Throwing and Catching a Ball; TGMD=Test of Gross Motor Development
